# Integrative Analysis of Sex-Specific microRNA Networks Following Stress in Mouse Nucleus Accumbens

**DOI:** 10.3389/fnmol.2016.00144

**Published:** 2016-12-23

**Authors:** Madeline L. Pfau, Immanuel Purushothaman, Jian Feng, Sam A. Golden, Hossein Aleyasin, Zachary S. Lorsch, Hannah M. Cates, Meghan E. Flanigan, Caroline Menard, Mitra Heshmati, Zichen Wang, Avi Ma'ayan, Li Shen, Georgia E. Hodes, Scott J. Russo

**Affiliations:** ^1^Fishberg Department of Neuroscience and Friedman Brain Institute, Icahn School of Medicine at Mount SinaiNew York, NY, USA; ^2^Graduate School of Biomedical Sciences, Icahn School of Medicine at Mount SinaiNew York, NY, USA; ^3^Department of Pharmacology and Systems Therapeutics, BD2K-LINCS Data Coordination and Integration Center, Icahn School of Medicine at Mount SinaiNew York, NY, USA

**Keywords:** depression, stress, sex differences, RNA-Seq, microRNA, nucleus accumbens

## Abstract

Adult women are twice as likely as men to suffer from affective and anxiety disorders, although the mechanisms underlying heightened female stress susceptibility are incompletely understood. Recent findings in mouse Nucleus Accumbens (NAc) suggest a role for DNA methylation-driven sex differences in genome-wide transcriptional profiles. However, the role of another epigenetic process—microRNA (miR) regulation—has yet to be explored. We exposed male and female mice to Subchronic Variable Stress (SCVS), a stress paradigm that produces depression-like behavior in female, but not male, mice, and performed next generation mRNA and miR sequencing on NAc tissue. We applied a combination of differential expression, miR-mRNA network and functional enrichment analyses to characterize the transcriptional and post-transcriptional landscape of sex differences in NAc stress response. We find that male and female mice exhibit largely non-overlapping miR and mRNA profiles following SCVS. The two sexes also show enrichment of different molecular pathways and functions. Collectively, our results suggest that males and females mount fundamentally different transcriptional and post-transcriptional responses to SCVS and engage sex-specific molecular processes following stress. These findings have implications for the pathophysiology and treatment of stress-related disorders in women.

## Introduction

Across cultures, adult women are twice as likely as men to suffer from affective and anxiety disorders including Major Depressive Disorder (MDD, Kessler et al., 1994, 2005; Bebbington, 1998). This enhanced female susceptibility begins in adolescence, declines post menopause, and is most pronounced during periods of great hormonal flux (perimenopause, postpartum period, late luteal phase), suggesting a role for female sex hormones in depression (Deecher et al., 2008; Gobinath et al., 2014). Once symptomatic, men and women experience depression differently—men are more likely to report anger, substance abuse, and risk-taking behavior; whereas women are more likely to experience somatic symptoms including irritability, fatigue, anhedonia, and sleep and appetite disturbances (Silverstein, 2002; Martin et al., 2013). Furthermore, women display enhanced risk of comorbid anxiety disorders and pain (Silverstein, 2002; Gobinath et al., 2014). Several studies suggest that sex differences may extend to treatment response, with women responding preferentially to selective serotonin reuptake inhibitors (SSRIs) over tricyclic antidepressants (TCAs), and men showing either no preference or better response to TCAs vs. SSRIs (Kornstein et al., 2000; Martenyi et al., 2001; Baca et al., 2004; Keers and Aitchison, 2010). Despite sex differences in MDD prevalence, symptoms, and treatment response, female subjects remain underrepresented in basic research, particularly in neuroscience (Beery and Zucker, 2011; Clayton and Collins, 2014).

Nevertheless, findings in animal models have yielded insight into the sexual dimorphism of MDD (Pfau and Russo, 2015). Stress is a key precipitating factor in the development of depressed mood and anxiety in human patients (Hammen, 2016; Pemberton and Fuller Tyszkiewicz, 2016). While adult female rodents often display greater cognitive resilience to chronic stress than males (Bowman et al., 2001; Luine, 2002; Conrad et al., 2003; Kitraki et al., 2004), they show enhanced emotional susceptibility to numerous stress paradigms (Westenbroek et al., 2003; Dalla et al., 2008; LaPlant et al., 2009; Sachs et al., 2014; Hodes et al., 2015a). Sex differences in the ventral striatum are increasingly implicated in adult female emotional vulnerability. The nucleus accumbens (NAc), a component structure of the ventral striatum, is essential for reward and emotion processing, and receives dense innervation from mood-related structures including the ventral tegmental area, amygdala, hippocampus, prefrontal cortex, and hypothalamus (Russo and Nestler, 2013). Recent studies highlight a role for NAc transcriptional and epigenetic processes in sex-specific stress responses. Following exposure to Subchronic Variable Stress (SCVS)—a stress paradigm that is sufficient to induce depression-like behavior in female mice but not in male mice—males and females exhibit remarkably different NAc transcriptional profiles that have been linked to both Nuclear factor κB (NfκB) transcription factor signaling (LaPlant et al., 2009) and epigenetic regulation by DNA methyltransferase 3a (Dnmt3a) (Hodes et al., 2015a).

Another potential epigenetic mechanism contributing to marked sex differences in stress-induced transcriptional patterns is post-transcriptional regulation by microRNAs (miRs). miRs are small, endogenous RNAs approximately 22 nucleotides in length. miRs do not encode proteins, but instead act to post-transcriptionally regulate the expression of target mRNA through sequence-specific binding, leading to subsequent mRNA destabilization or translational repression (Bartel, 2004; O'Carroll and Schaefer, 2013; Eichhorn et al., 2014). The evolutionary potency of miRs underscores their importance, and indeed, bioinformatic predictions estimate that 30% of mammalian protein-coding mRNAs are subject to miR regulation (O'Connor et al., 2012). Although the effect on protein expression of a single miR-mRNA interaction is modest, miRs can have hundreds of targets, and multiple miRs can target the same mRNA simultaneously (O'Carroll and Schaefer, 2013). miRs are highly enriched in the brain—50% of known mammalian miRs are expressed in the brain—and have been implicated in numerous neural processes including circuit formation and plasticity; synaptic function; and neuronal survival, differentiation, and diversity (O'Carroll and Schaefer, 2013). Dysregulated miRs have been reported in postmortem brain (Smalheiser et al., 2012; Issler et al., 2014; Lopez et al., 2014a,b; Maheu et al., 2015), blood (Belzeaux et al., 2012; Li et al., 2013; Fan et al., 2014; Issler et al., 2014; Lopez et al., 2014a; Camkurt et al., 2015), and dermal fibroblasts (Garbett et al., 2015) of depressed patients. In preclinical models, miRs have been implicated in depression-related processes ranging from glucocorticoid resistance (Uchida et al., 2008; Vreugdenhil et al., 2009) and corticotropin releasing factor sensitivity (Haramati et al., 2011), to behavioral resilience (Smalheiser et al., 2011; Dias et al., 2014; Issler et al., 2014) and antidepressant efficacy (Baudry et al., 2010; Issler et al., 2014).

In the present study, we aimed to characterize the NAc transcriptional and post-transcriptional landscape associated with sex differences in behavioral response to SCVS. Using RNA Sequencing (RNA-Seq), we performed an unbiased, genome-wide bioinformatic analysis of sex-specific miR and mRNA transcriptome profiles in mouse NAc following exposure to SCVS. We created miR-mRNA networks to probe and illustrate the complexity of stress-induced miR regulation in males and females. We find that male and female mice initiate fundamentally different transcriptional and post-transcriptional responses to stress. Furthermore, we report that male mice are not insensitive to SCVS, but rather mount a consistently robust transcriptional and post-transcriptional response to stress. This study provides new insight into the multiple levels of transcriptional processes that inform sex differences in stress susceptibility.

## Materials and methods

### Animals

C57BL/6J male and female mice (Jackson Laboratory, Bar Harbor, ME) aged 8 weeks were used for all RNA-Seq and quantitative real-time PCR (qPCR) validation studies. All mice were shipped to the Icahn School of Medicine at Mount Sinai (ISMMS) animal facility at 7 weeks of age and were acclimated to the facility for 1 week prior to SCVS. Mice were group housed and maintained on a 12-h light/dark cycle in which lights were on between 7 A.M. and 7 P.M. with *ad libitum* access to food and water. All mouse procedures were approved by and performed in accordance with the National Institute of Health Guide for Care and Use of Laboratory Animals and the Institutional Animal Care and Use Committee at the Icahn School of Medicine at Mount Sinai. Different mouse cohorts were used for RNA-Seq and qPCR validation studies.

### Subchronic variable stress (SCVS)

SCVS, which consists of three stressors administered over the course of 6 days, was performed as described previously (LaPlant et al., 2009; Hodes et al., 2015a). One unpredictable stressor was administered for an hour each day, and the stressors were alternated during the 6 days to prevent habituation. The three stressors included, in order: foot shock, tail suspension, and physical restraint. For foot shock stress, 10 same sex mice were placed in a fear conditioning chamber with electric grid flooring (Med Associates, St. Albans, VT) and administered 100 random 0.45 mA, 2 s foot shocks. For tail suspension stress, mouse tails were adhered to a bar, leaving the mice suspended for 1 h in an inverted position. For physical restraint stress, mice were placed in a well-ventilated, 50-mL centrifuge tube in the home cage for 1 h. The stressors were repeated in the same order on days 4–6, such that mice received foot shock stress on days 1 and 4, tail suspension stress on days 2 and 5, and restraint stress on days 3 and 6. After each stressor, mice were returned to the home cage until the next stressor or sacrifice. Unstressed controls were group housed in the home cage until sacrifice. SCVS-induced depression-like behavior was not assayed prior to sacrifice to avoid the introduction of testing-induced transcriptional changes. Furthermore, post-SCVS behavioral phenotypes have been well documented previously (LaPlant et al., 2009; Hodes et al., 2015a). The behavioral endpoints of SCVS in female mice include decreased sucrose preference (Hodes et al., 2015a), increased latency to eat in the novelty suppressed feeding test (Hodes et al., 2015a), increased immobility in the forced swim test (LaPlant et al., 2009; Hodes et al., 2015a), and increased latency to groom in the splash test (Hodes et al., 2015a). In contrast, male mice behave similarly to unstressed controls across these behavioral measures (LaPlant et al., 2009; Hodes et al., 2015a).

### Estrous cycle monitoring

Vaginal lavage was performed on all female animals at sacrifice to determine estrous cycle stage. A sterile cotton swab moistened with 0.9% saline was used to collect a sample of vaginal cells from each animal. The sample was smeared onto a slide, left to dry overnight, and stained with 1% Toluidine Blue (Sigma-Aldrich, St. Louis, MO). Cycle stage was determined by visual inspection of cell morphology under a light microscope and recorded for each animal. Lavage was only performed at the point of sacrifice to best facilitate direct comparison of the sexes. Repeated lavage affects reward-related processes such as cocaine-stimulated motor behavior, and lavaged rats develop a conditioned place preference to a lavage-paired compartment (Walker et al., 2002). Therefore, daily lavage across all 6 days of the stress and at sacrifice could be potentially confounding. Furthermore, in an independent cohort of mice, we found that female mice cycled normally throughout exposure to the stress protocol (Figure S1) and did not demonstrate a stress-induced shift in length and duration of the estrous cycle that has been reported for other chronic stress paradigms (Konkle et al., 2003; Tou et al., 2004).

### Tissue collection, RNA extraction, and cDNA synthesis

NAc samples were collected from each animal as described previously (Golden et al., 2013; Hodes et al., 2015a). Twenty-four hours after the last stressor, animals were rapidly decapitated and bilateral 14-gauge ventral striatum punches were collected on ice. Punches were flash frozen on dry ice and stored at −80°C until RNA extraction. RNA was isolated using homogenization in Qiazol (Qiagen, Hilden, Germany) followed by chloroform layer separation. For sequencing, bilateral NAc punches from 5 animals (10 NAc punches) were pooled for each library at the point of homogenization. Pooling was necessary to yield sufficient input for miR sequencing, and was controlled for estrous cycle stage such that all female samples were derived from mice in estrus and proestrus. Mice in diestrus and metestrus were excluded from analysis. The aqueous RNA layer was further processed via miRNeasy mini kit (Qiagen, Hilden, Germany) to yield separate fractions enriched for small RNA (<200 bp; for small RNA sequencing) and large RNA (>200 bp; for mRNA sequencing). RNA and miR quality was assessed by Bioanalyzer (Agilent, Santa Clara, California). For qPCR validations, total RNA was extracted and purified (miRNeasy micro kit, Qiagen, Hilden, Germany) from bilateral NAc punches from individual animals. The RNA was then analyzed by Nanodrop (Thermo Fisher Scientific, Waltham, MA), and 500 ng of RNA was reverse transcribed to cDNA (qScript cDNA Supermix, Quanta Biosciences, Beverly, MA). cDNA was diluted in nuclease-free water to 500 uL (1 ng/uL).

### Small RNA sequencing and differential analysis

Small RNA-Seq libraries were generated from enriched small RNA fractions (<200 bp) according to the instructions of the Scriptminer Small RNA-Seq Library Preparation Kit (Epicentre, Madison, WI) with modifications as described in Dias et al. (2014). Libraries were constructed from pooled RNA samples (*n* = 5 mice/library) and run in independent biological replicates of 3 libraries per sex/stress condition for a total of 12 libraries. Library size and concentration were confirmed by Bioanalyzer (Agilent, Santa Clara, CA) prior to sequencing. Multiplexed libraries were pooled 12 to a lane and sequenced on a single lane of a HiSeq 2000 sequencer (Illumina, San Diego, CA) with 100 bp single-end reads at the ISMMS Genomics Core. Raw sequencing reads were processed by fastx trimmer (http://hannonlab.cshl.edu/fastx_toolkit) to remove the 3′ adaptor sequence, and sequences shorter than 16 nucleotides were subsequently discarded. Sequencing quality was assessed by FastQC (http://www.bioinformatics.babraham.ac.uk/projects/fastqc). Sequencing yielded 192 million reads, with approximately 16 million high quality reads per sample. Reads were aligned to the mm9 mouse genome using Bowtie short read aligner (Langmead et al., 2009) and quantified against mature miRBase release 21 annotation using the HT-Seq Python package (Anders et al., 2015). Differential analysis was performed using the voom-limma R package (Law et al., 2014; Ritchie et al., 2015). A multifactorial, gene-wise linear model with stress and sex as main factors was fit to expression data to determine the effect of stress in males and females separately and to control for baseline variation in miR expression. An uncorrected *p* < 0.05 and a fold change threshold of 1.3 were used to determine differential miR expression, in accordance with the statistical cutoff used for NAc small RNA-Seq in Dias et al. (2014). Small RNA-Seq data has been made publicly available in the Gene Expression Omnibus (GEO) repository under the accession number GSE90962.

### mRNA sequencing and differential analysis

RNA-Seq libraries were generated from large RNA fractions (>200 bp) with RIN values > 8.8 as described in Feng et al. (2014). As for small RNA sequencing, libraries were constructed from pooled RNA samples (*n* = 5 mice/library) and run in independent biological replicates of 3 libraries per sex/stress condition for a total of 12 libraries. RNA (1 ug) was used for mRNA library construction according to the instructions of the TruSeq RNA Library Preparation Kit v2 (Illumina, San Diego, CA) as described in Hodes et al. (2015a). Libraries were evaluated by Bioanalyzer (Agilent, Santa Clara, CA) to verify size and concentration before pooling 12 to a lane and sequencing on 3 lanes of a HiSeq 2000 machine (Illumina, San Diego, CA) with 50 bp paired-end reads at the ISMMS Genomics Core. Sequencing yielded >30 million high quality reads per sample. Short reads were aligned to the Ensembl *mus musculus* NCBIM37 version 67 reference genome using TopHat2 (Kim et al., 2013). Quality assessment revealed a >90% mapping rate, with ~10% of those reads mapping to mitochondrial RNA and <1% aligning to ribosomal RNA. Individual gene counts for each sample were obtained using the HTSeq Python package (Anders et al., 2015). Only genes with >5 reads in at least 80% of samples in any one of the experimental conditions were included in differential analysis. Differential analysis was performed using the voom-limma R package (Law et al., 2014; Ritchie et al., 2015). A multifactorial, gene-wise linear model with stress and sex as main factors was fit to expression data to determine the effect of stress in males and females separately and to control for baseline variation in gene expression. An uncorrected *p* < 0.05 and a fold change threshold of 1.3 were used to determine differential gene expression, in accordance with the statistical cutoff used for NAc RNA-Seq in previously published reports (Hodes et al., 2015a; Bagot et al., 2016). RNA-Seq data has been made publicly available in the Gene Expression Omnibus (GEO) repository under the accession number GSE90962.

### Quantitative overlap

Quantitative overlap analysis was performed as described in Hodes et al. (2015a) using the *SuperExactTest* R software package (Wang et al., 2015). Differentially expressed mRNAs or miRs were divided into upregulated and downregulated lists. *SuperExactTest* R software was utilized to compare lists pairwise, generating 22 unique comparisons. mRNA/miR identifiers from each list were extracted for each comparison, and the quantitative overlap for each list was determined and statistically analyzed via a Fisher's Exact test. For all comparisons, significance was set at *p* < 0.05 and the number of background mRNAs/miRs was kept constant to reflect the total number of candidate mRNAs/miRs analyzed in the differential expression analysis (19,827 mRNAs, 781 miRs).

### miR-mRNA network analysis

Predicted targets of stress-regulated miRs in males and females were identified using miRWalk 2.0 (http://zmf.umm.uni-heidelberg.de/apps/zmf/mirwalk2/), an online archive of predicted and experimentally validated miR-target interactions (Dweep et al., 2011; Dweep and Gretz, 2015). miRWalk searches were customized to yield only target mRNAs predicted by both the TargetScan and miRWalk algorithms for subsequent network analysis. Significant association of predicted targets of stress-regulated miRs within mRNA differential lists was assessed using chi-squared tests. The frequencies of upregulated, downregulated, and neutral mRNAs in stressed vs. control groups were computed from mRNA lists for each sex and were used as background. Then, for each differentially regulated miR, the frequencies of upregulated, downregulated and neutral *target* genes were computed from the mRNA differential list referencing the miRWalk predicted target list. A chi-squared test was then performed for each miR, comparing the frequencies of up, down and neutral *target* mRNAs versus that of the background. Significant miRs surviving this analysis were identified using a Benjamini-Hochberg adjusted *p*-value cut-off of *p* < 0.05. miR-mRNA relationships were then classified as having a positive association (both the miR and target mRNA are regulated by stress in the same direction) or a negative association (the miR and target mRNA are regulated by stress in opposite directions). miR-target network visualizations were constructed using Cytoscape v3.3.0 (cytoscape.org).

### Functional annotation and pathway analysis

Canonical Pathways and Diseases and Functions analyses were performed on differentially regulated genes and miR-targeted genes using Qiagen's Ingenuity Pathway Analysis (IPA, Qiagen Redwood City, CA, http://www.qiagen.com/ingenuity). Gene Ontology (GO) analysis was performed on differentially regulated and miR-targeted genes with Database for Annotation, Visualization and Integration Discovery (DAVID) Bioinformatics Resources (https://david.ncifcrf.gov/) using the DAVID *mus musculus* background (Huang da et al., 2009a, 2009b). IPA pathways/functions and GO terms were considered significantly enriched when Fisher's Exact –log(*p*-value) ≥ 1.3.

### Quantitative real-time PCR

Quantitative real-time PCR (qPCR) biological validations of RNA-Seq targets were performed on NAc samples from individual animals. The qPCR reaction mixture consisted of 5 uL PerfeCta SYBR Green SuperMix, ROX (Quanta Biosciences, Beverly, MA), 1 uL primer (PrimeTime predesigned qPCR primers, Integrated DNA Technologies, Coralville, IA), 1 uL nuclease-free water, and 3 uL cDNA template. Samples were heated to 95°C for 2 min followed by 40 cycles of 95°C for 15 s, 60°C for 33 s, and 72°C for 33 s on an Applied Biosystems 7900 RT PCR System (Foster City, CA). Data were analyzed using the 2^−ΔΔ(Ct)^ method (Livak and Schmittgen, 2001). Expression values were normalized to stably expressed reference genes (*Lsm4, Kcnc2*) that were selected from RNA-Seq gene lists based on their lack of regulation by SCVS. Expression values were normalized such that control groups show no fold change.

### Statistical analysis

For qPCR validations, differences between stressed and unstressed groups were compared using unpaired *t*-tests (two-tailed) or Wilcoxon Mann-Whitney (two-tailed) nonparametric *U*-tests when group variances were unequal. All statistical analyses were performed using GraphPad Prism 5.0 software (GraphPad Software Inc., La Jolla, CA). Statistical significance was set at *p* < 0.05. Grubbs outlier test was performed and samples that varied more than two standard deviations from the mean were removed.

## Results

### SCVS induces largely non-overlapping mir transcriptional profiles in male and female mice

**Figure 1:**
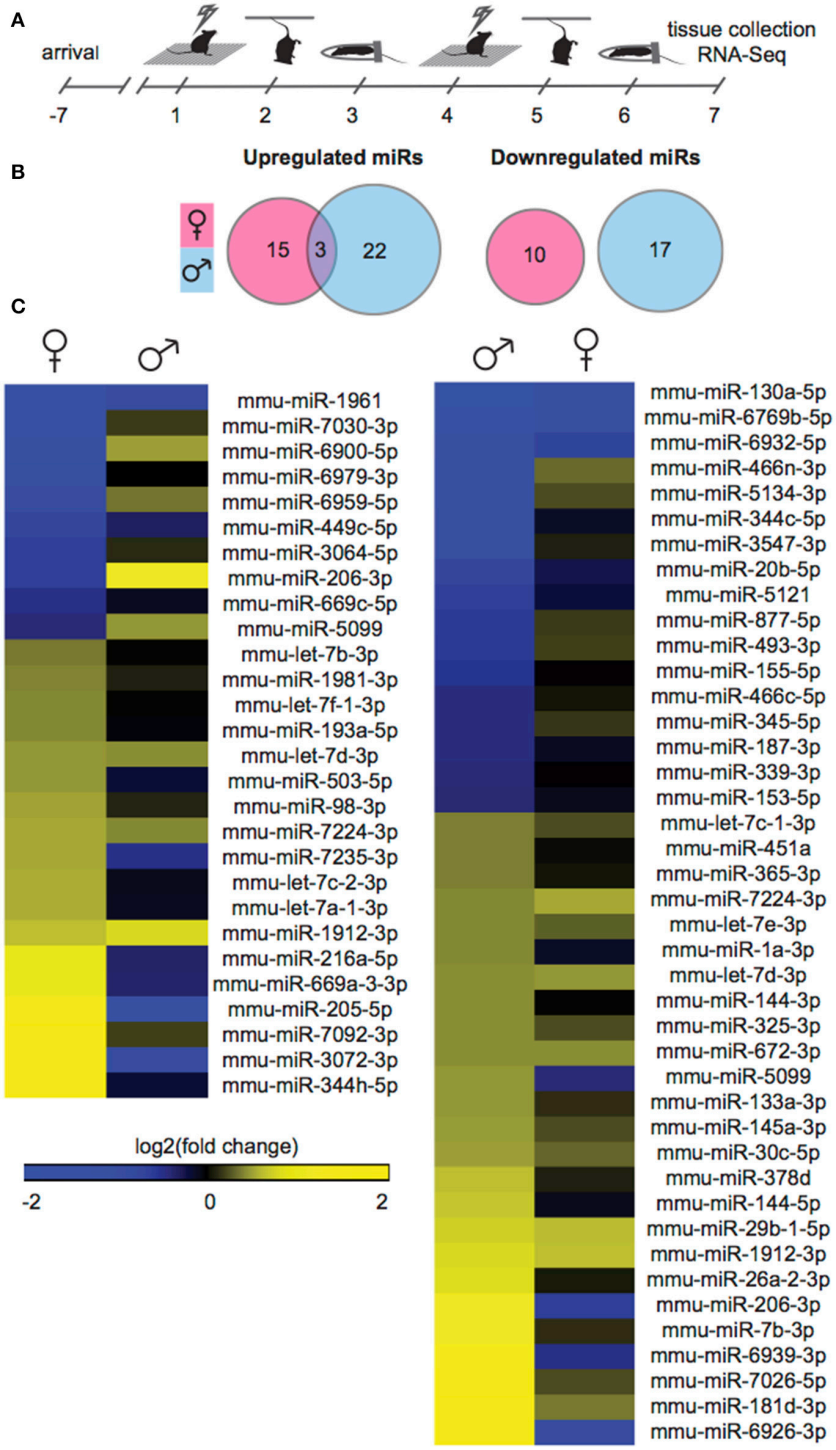
**Male and female mice show different NAc miR profiles following Subchronic Variable Stress. (A)** Schematic of the Subchronic Variable Stress (SCVS) paradigm. **(B)** Union maps of significantly upregulated (Left) and downregulated (Right) microRNAs (miRs) in males and females following SCVS. **(C)** (Left) Heatmap of miRs differentially regulated by SCVS in females compared to the same miRs in males, (Right) Heatmap of miRs differentially regulated by SCVS in males compared to the same miRs in females. Yellow indicates upregulation, blue indicates downregulation, and black indicates no change.

Gonadally intact, adult male and female mice were exposed to SCVS, consisting of 6 consecutive days of 3 alternating, unpredictable stressors: repeated foot shock, tail suspension, and physical restraint (Figure 1A). To profile stress-induced changes to the global NAc miRnome, we performed small RNA-Seq on NAc tissue collected 24 h after the last stressor. We compared stressed groups to same sex, unstressed controls in order to identify sex-specific patterns. Importantly, we controlled for female estrous cycle by including only females in estrus or proestrus so as to capture a period of great hormonal flux in estrogen and progesterone levels (Scharfman and MacLusky, 2008), and therefore recapitulate the peak sensitivity of female rodents to stress (Viau and Meaney, 1991; Carey et al., 1995; Bangasser and Valentino, 2014) and women to depression (Hendrick et al., 1998; Cohen et al., 2006; Gobinath et al., 2014). In males, 42 miRs were regulated by SCVS compared to unstressed male controls: 25 miRs were upregulated, and 17 miRs were downregulated (Figure 1B, Table S2). In females, 20% fewer miRs—28 miRs—were regulated by SCVS: 18 miRs were upregulated, and 10 miRs were downregulated (Figure 1B, Table S2). As illustrated by union maps (Figure 1B) and heatmap visualizations (Figure 1C), miR transcriptional profiles for males and females were largely non-overlapping. We submitted male and female miR differential lists to quantitative overlap analysis, and found that male and female miRnome profiles showed significant quantitative overlap only in upregulated miRs (mmu-miR-7224-3p, mmu-let-7d-3p, mmu-miR-1912-3p; *p* < 0.02) and oppositely regulated miRs (mmu-miR-5099, mmu-miR-206-3p; *p* < 0.04).

### Males mount a robust, sex-specific transcriptional response to SCVS

**Figure 2:**
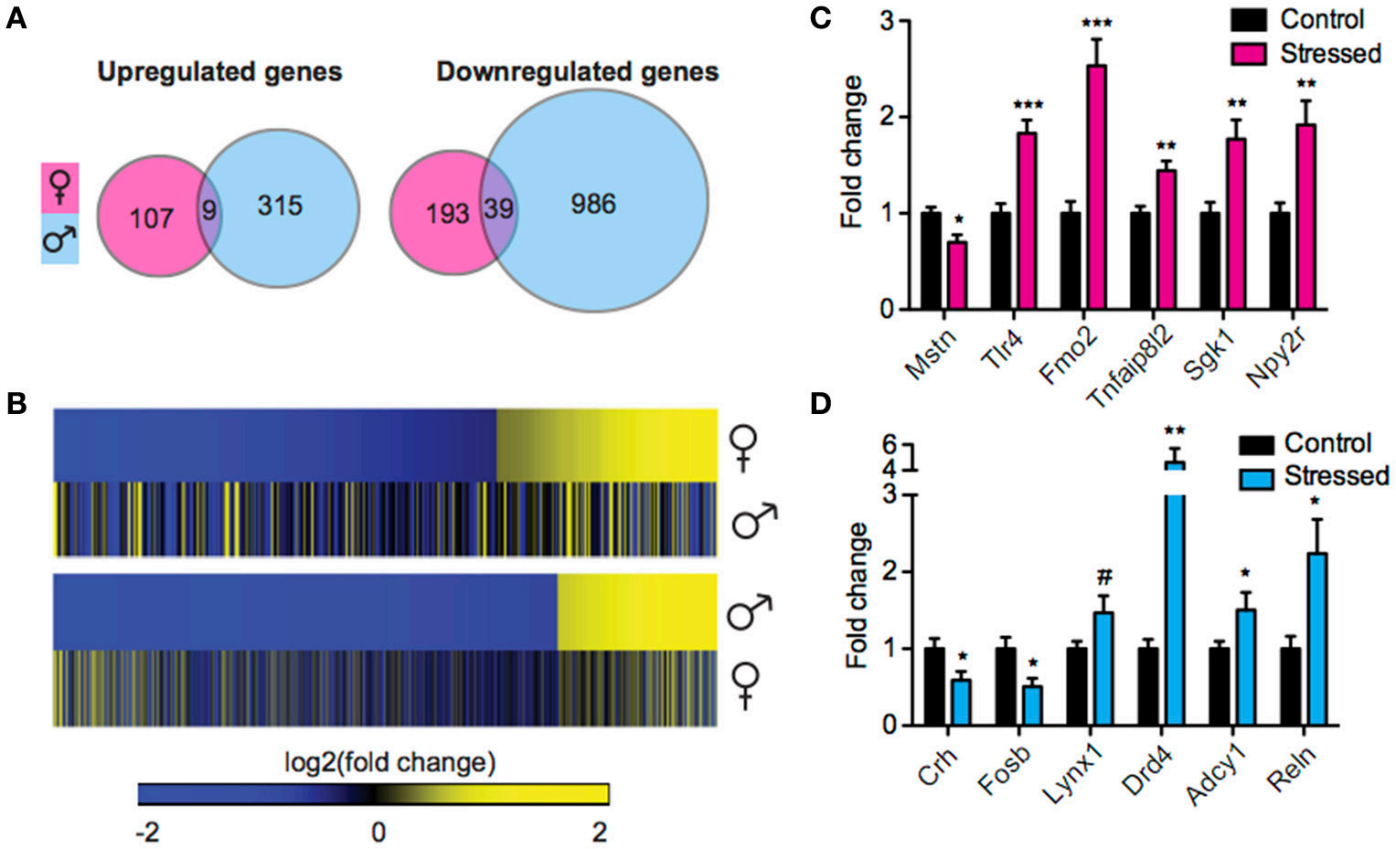
**Male and female mice show different NAc mRNA profiles following Subchronic Variable Stress. (A)** Union maps of significantly upregulated (Left) and downregulated (Right) genes in males and females following Subchronic Variable Stress (SCVS). **(B)** (Top) Heatmap of genes differentially regulated by SCVS in females compared with the same genes in males, (Bottom) Heatmap of genes differentially regulated by SCVS in males compared with the same genes in females. Yellow indicates upregulation, blue indicates downregulation, and black indicates no change. **(C)** Validation by quantitative real-time PCR (qPCR) of a subset of genes regulated by SCVS in females (*n* = 9–11 per group). **(D)** Validation by qPCR of a subset of genes regulated by SCVS in males (*n* = 9–11 per group). ^*^p < 0.05, ^**^p < 0.01, ^***^p < 0.001, ^#^*p* < 0.1.

We next sought to interrogate the effect of SCVS on gene expression in the same animals by performing RNA-Seq. RNA-Seq allowed us to quantify the expression levels of all polyA-containing transcripts in stressed mice and controls. In stressed males compared to unstressed control males, 1349 genes were regulated by SCVS (Figure 2A, See Table S3 for top 20 up and downregulated genes and Data Sheet 2 for full list). The majority of stress-regulated genes in males were downregulated (~76% or 1025 genes, vs. 324 upregulated genes). Strikingly, despite the insufficiency of SCVS to induce behavioral and neuroendocrine changes in male mice, males demonstrated a very robust transcriptional response to SCVS, potentially indicating active resilience mechanisms. In stressed females compared to unstressed control females, 348 genes were regulated by SCVS (Figure 2A, See Table S3 for top 20 up and downregulated genes and Data Sheet 2 for full list). As in males, the majority of stress-regulated genes in females were downregulated (~67% or 232 genes, vs. 116 upregulated genes). Similar to what we observed for miRs, male and female SCVS-induced genome profiles showed little overlap—approximately 2% of all upregulated genes (9 genes, *p* = 1.23E^−4^) and ~3% of all downregulated genes (39 genes, *p* = 6.58E^−11^) (Figures 2A,B). In total, 21 genes were regulated in opposite directions. Twelve genes were upregulated in males and downregulated in females (*p* = 4.58E^−4^): *Plekha2, Etl4, Chrna4, AC154308.1, St14, Fhl2, Alas2, Arhgap15, Adam18, AC133502.1, Rhox4a*, and *Chrnb3*. Nine genes were downregulated in males and upregulated in females (*p* = 0.15): *AL671140.1, Tex16, Gm12494, Nts, U6, Arhgap36, Ropn1l, Tnfsf9*, and *Fas*. As shown in Figures 2C,D, we performed biological validations of several genes by qPCR: *Mstn* [*t*_(17)_ = 2.86, *p* = 0.01], *Tlr4* [*t*_(18)_ = 4.93, *p* < 0.001], *Fmo2* (*U* = 3.00, *p* < 0.001), *Tnfaip8l2* [*t*_(19)_ = 3.65, *p* < 0.01], *Sgk1* [*t*_(19)_ = 3.46, *p* < 0.01], *Npy2r* (*U* = 10.00, *p* < 0.01), *Crh* [*t*_(18)_ = 2.25, *p* = 0.04], *Fosb* [*t*_(18)_ = 2.68, *p* = 0.02], *Lynx1* (*U* = 24.00, *p* = 0.09), *Drd4* (*U* = 13.00, *p* < 0.01), *Adcy1* (*U* = 23.00, *p* = 0.048), and *Reln* (*U* = 17.00, *p* = 0.02). Genes for validation were chosen from IPA output for their involvement in significantly enriched functional networks and pathways.

### Functional ontology and pathway analysis reveals sex differences in stress-induced functional processes

**Figure 3:**
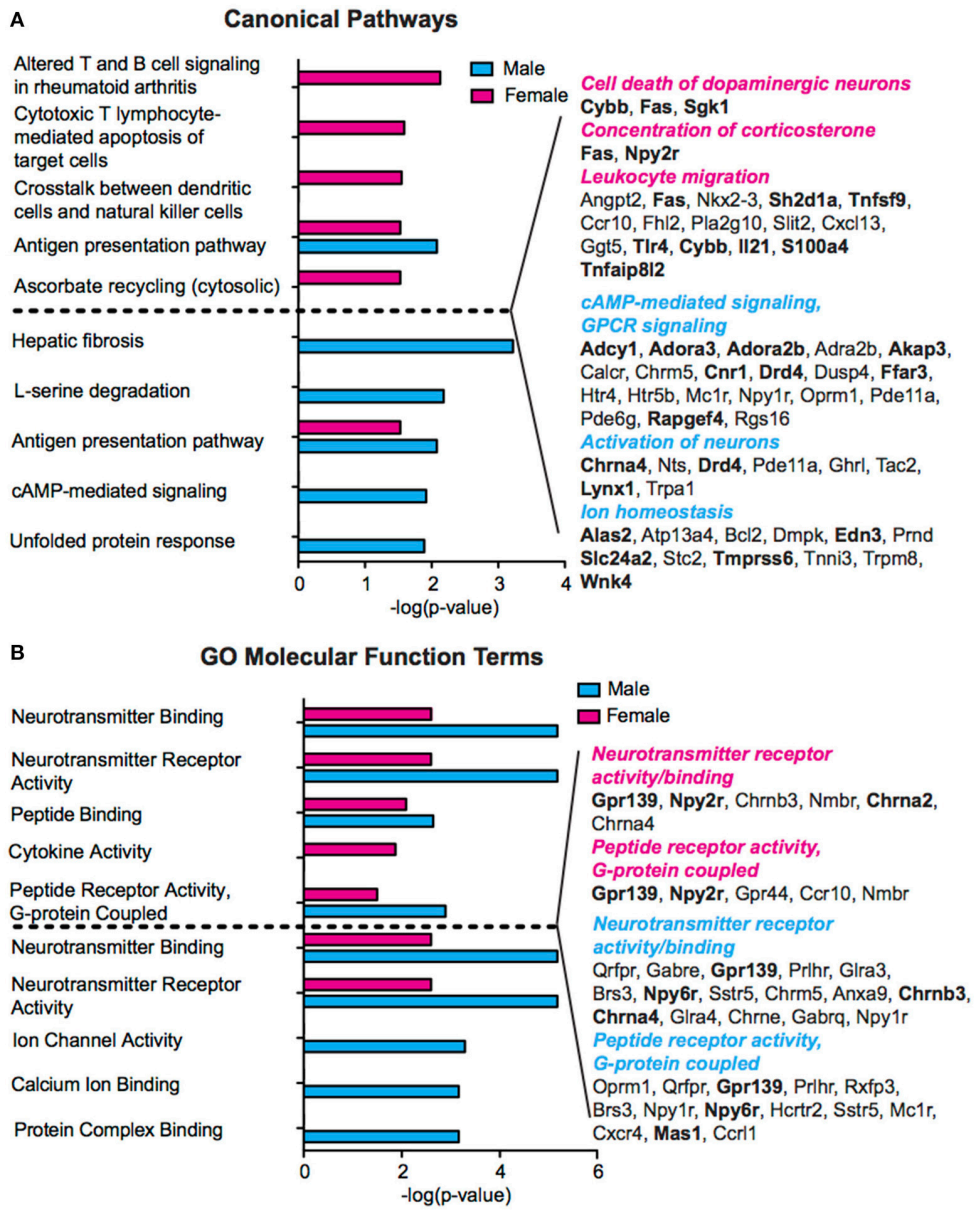
**Sex differences in pathway enrichment and gene ontology of genes regulated by Subchronic Variable Stress. (A)** (Left) The top five significantly enriched (*p* < 0.05) IPA canonical pathways in females (top, magenta) and males (bottom, blue), shown as –log(*p*-value). For pathways significantly enriched in both sexes, the degree of enrichment of the pathway in the opposite sex is shown in a separate, immediately adjacent bar. (Right) Hand curated list of genes relevant to depression pathology chosen from significantly enriched IPA Canonical Pathways or from within the top 50 significantly enriched IPA Diseases and Functions. Upregulated genes are shown in bold font. Enriched pathways/functions in females are colored magenta whereas those for males are colored blue. **(B)** (Left) The top five significantly enriched (*p* < 0.05) GO Molecular Functions in females (top, magenta) and males (bottom, blue) as identified by DAVID, shown as–log(*p*-value). For GO terms significantly enriched in both sexes, the degree of enrichment of the term in the opposite sex is shown in a separate, immediately adjacent bar. (Right) Genes corresponding to the neurotransmitter receptor activity and binding, and peptide receptor activity, G-protein coupled, molecular functions in females (top) and males (bottom), with upregulated genes shown in bold font.

We performed pathway and gene ontology (GO) analysis on our RNA-Seq mRNA differential lists using IPA and DAVID tools, respectively (Figure 3, Table S4). We utilized both tools to capitalize on their respective strengths—IPA draws from a curated, proprietary knowledgebase of published, experimentally validated information, while DAVID utilizes a comprehensive knowledgebase compiled from dozens of publicly available bio-databases (BioCarta, KEGG, GO, etc.) to maximize analytic power. Reflecting our finding of minimally overlapping SCVS-induced genes, we observed enrichment of different functional processes in males and females. Only one canonical pathway—Antigen presentation pathway, which encompasses antigen recognition and processing essential for innate and adaptive immunity—is represented among significantly enriched canonical pathways in both males and females (Figure 3A, Table S3). Among significantly enriched DAVID GO molecular function terms for males and females, we observed overlap of five functions—Neurotransmitter receptor activity and binding, peptide receptor activity and binding, and G-protein coupled peptide receptor activity—however, different or, in some cases, oppositely regulated genes were enriched within these pathways in the two sexes (Figure 3B, Table S3). Overall, we observed enrichment of several processes relevant to stress and depression, including inflammatory processes and cytokine activity (Hodes et al., 2014, 2015b), cAMP and GPCR-mediated signaling (Duman et al., 1999; Terzi et al., 2009) and ion channel activity (Friedman et al., 2014). Importantly, we observed overlap of numerous functional categories between our IPA and DAVID analyses, with terms including ion homeostasis/channel activity and GPCR signaling appearing in both analyses for males, and inflammatory processes appearing in both analyses for females.

### SCVS induces sex dependent miR-mRNA networks

**Figure 4:**
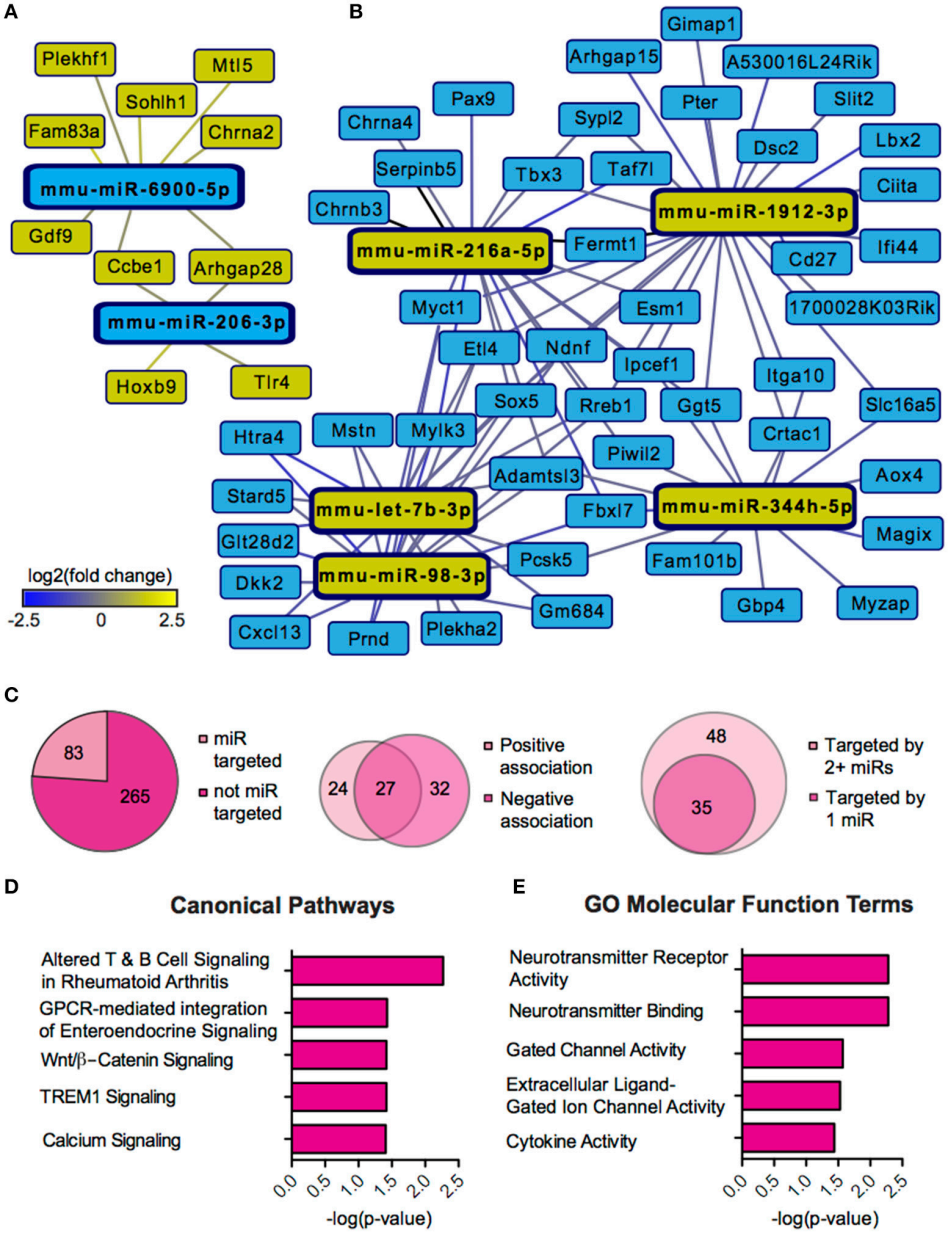
**Female miR-mRNA networks regulated by Subchronic Variable Stress. (A)** Networks of downregulated microRNAs (miRs, blue nodes) surviving network analysis and their negatively associated, upregulated target genes (yellow nodes). **(B)** Networks of upregulated miRs (yellow nodes) surviving network analysis and their negatively associated, downregulated target genes (blue nodes). Blue lines indicate target gene downregulation whereas yellow lines indicate target gene upregulation. Line brightness illustrates the degree of up/downregulation, with brighter lines connecting to more strongly regulated genes. **(C)** Pie charts and union maps illustrating genes targeted and not targeted by miRs (Left), genes showing a negative and/or positive association with targeting miRs (Middle), and genes targeted by 1 miR or 2+ miRs (Right). **(D)** The top five significantly enriched (*p* < 0.05) IPA canonical pathways for female miR-targeted genes. **(E)** The top five significantly enriched (*p* < 0.05) GO Molecular Functions for female miR-targeted genes as identified by DAVID.

**Figure 5:**
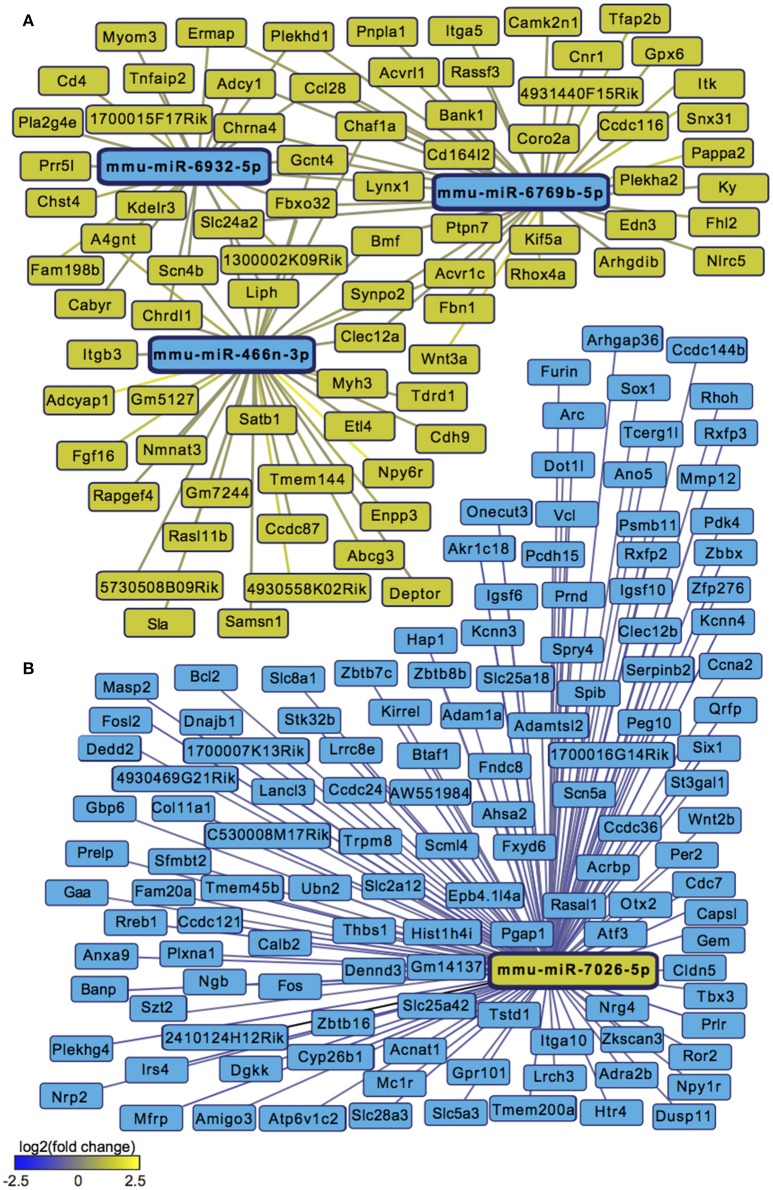
**Male miR-mRNA networks regulated by Subchronic Variable Stress. (A)** Networks of the top three most strongly downregulated miRs (blue nodes) surviving network analysis and their negatively associated, upregulated target genes (yellow nodes). **(B)** Network of one of the top three most strongly upregulated miRs (yellow node) surviving network analysis and its negatively associated, downregulated target genes (blue nodes). Blue lines indicate target gene downregulation whereas yellow lines indicate target gene upregulation. Line brightness illustrates the degree of up/downregulation, with brighter lines connecting to more strongly regulated genes.

**Figure 6:**
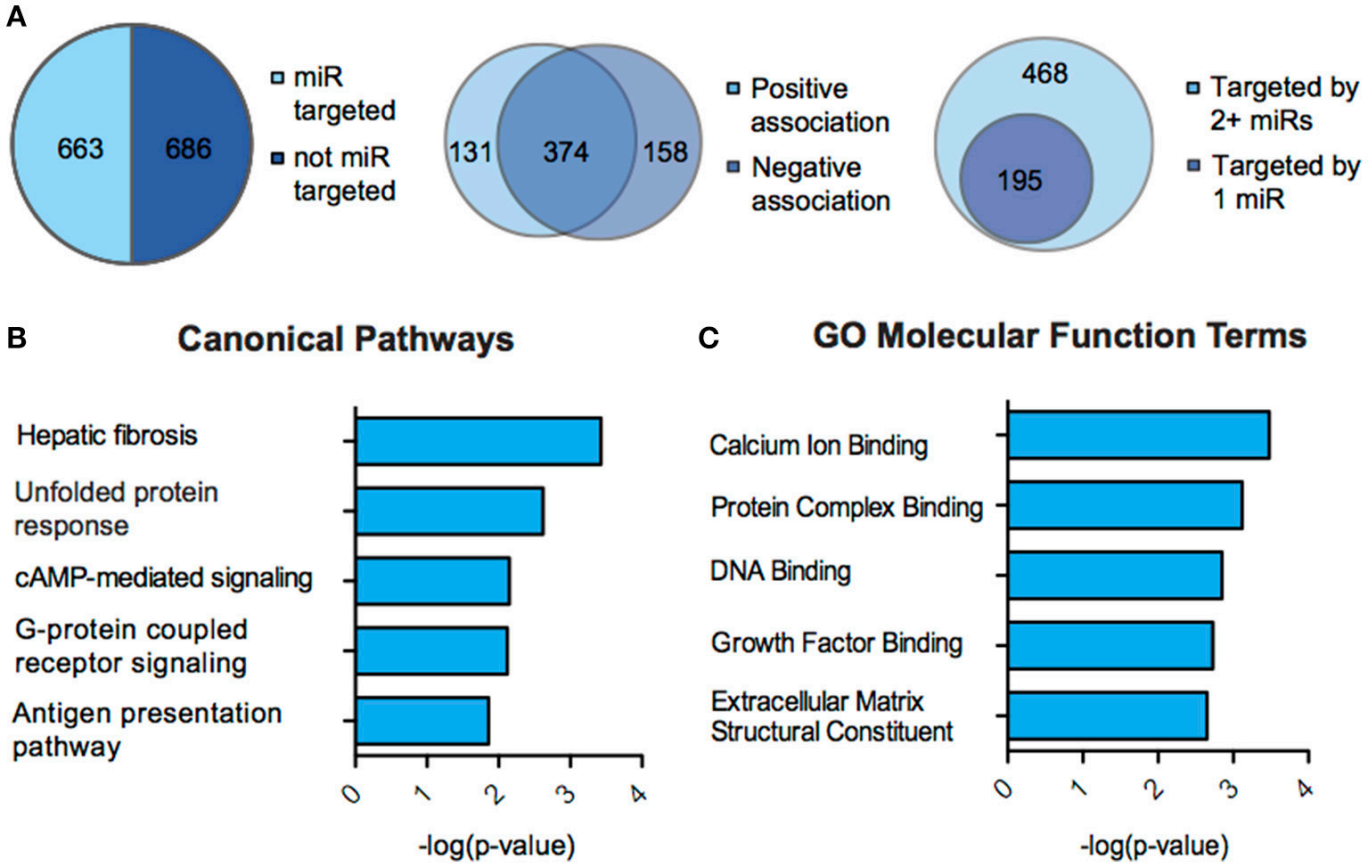
**miR-mRNA regulatory landscape and functional enrichment of miR-targeted genes in males. (A)** Pie charts and union maps illustrating genes targeted and not targeted by microRNAs (miRs, Left), genes showing a negative and/or positive association with targeting miRs (Middle), and genes targeted by 1 miR or 2+ miRs (Right). **(B)** The top five significantly enriched (*p* < 0.05) IPA canonical pathways for male miR-targeted genes. **(C)** The top five significantly enriched (*p* < 0.05) GO Molecular Functions for male miR-targeted genes as identified by DAVID.

We next sought to overlay miR and mRNA differential lists to probe the nature and complexity of miR-mRNA interactions in males and females. In order to accomplish this, we identified all mRNA targets of SCVS-regulated miRs predicted by both TargetScan and miRWalk online databases (Lewis et al., 2005). We then applied a novel chi-squared analysis to measure enrichment of predicted miR target genes in our RNA-Seq mRNA differential lists. In females, 7 miRs (25%) survived the analysis, two downregulated miRs and 5 upregulated miRs (Figures 4A,B, Table S5). Eighty-three genes, or ~24%, were miR-targeted (Figure 4C). For simplicity, we illustrated only miR target genes that satisfy the canonical negative miR-mRNA target association in network diagrams, however, ~29% of genes showed only a positive association with targeting miRs, and ~33% of genes showed both positive and negative associations (Figure 4C). The majority of target genes—48 genes or ~58%—were targeted by more than one miR, whereas 35 genes (~42%) were targeted by only one miR (Figure 4C). We performed IPA (Figure 4D, Table S8) and DAVID (Figure 4E, Table S8) analyses on miR-targeted genes, finding enrichment of several processes relevant to depression, including Wnt/ß-Catenin signaling (Dias et al., 2014), calcium signaling and immune processes (Hodes et al., 2014, 2015b). Interestingly, we observed overlap of significantly regulated canonical pathways (Altered T and B Cell Signaling in Rheumatoid Arthritis) and GO molecular function terms (Neurotransmitter receptor activity, binding; Cytokine activity) between analyses of all SCVS-regulated genes and miR-targeted genes, indicating an involvement of miRs in prominent stress-induced molecular processes. In males, 23 miRs (~55%) survived the analysis, 12 downregulated miRs and 11 upregulated miRs (Tables S6, S7). Due to the number and size of miR-mRNA networks in males, we illustrated only the networks corresponding to the top 3 miRs most strongly downregulated by SCVS and one of the top 3 miRs most strongly upregulated by SCVS (Figures 5A,B). The remaining miRs and targeted mRNAs are listed in Tables S6, S7. Again, we only included miR target genes with a negative association to the miR of interest in network illustrations. Six hundred sixty-three genes (~49%) were miR-targeted (Figure 6A). Of these, 532 genes (~80%) showed a canonical negative association with at least 1 miR, whereas 131 genes (~20%) showed only a positive association (Figure 6A). The majority of miR-targeted genes (468 genes, or ~71%) were targeted by more than 1 miR, whereas 195 genes (~29%) were targeted by only 1 miR (Figure 6A). IPA analysis on miR-targeted genes revealed that, of the 13 significantly enriched canonical pathways for the male miR-targeted gene list, 7 overlapped with those enriched in the SCVS male differential gene list (the top 5 enriched pathways are represented in Figure 6B, the full list is represented in Table S8). DAVID analysis revealed that 24 of the 35 significantly enriched GO molecular function terms in the miR-targeted gene list overlapped with those from the full SCVS list, further illustrating the potential involvement of miR regulation in stress responsive molecular processes (the top 5 enriched GO terms are represented in Figure 6C, the full list is represented in Table S8).

## Discussion

We find that SCVS produces striking sex differences in NAc transcriptional and post-transcriptional profiles. We observe male/female overlap of only ~3% of similarly regulated mRNAs and ~4% of similarly regulated miRs following SCVS. We also find minimal overlap between sexes in enriched functional processes. Rather than displaying a resistance to SCVS-induced transcriptional changes that mirrors the male behavioral response, males mount a robust transcriptional response to stress. This “active” response extends to male post-transcriptional profiles and miR-mRNA networks. These findings provide unique insight into the molecular underpinnings of sexual dimorphism in stress response.

The majority of studies investigating miR regulation of target mRNAs in psychiatric disease patients and animal models have used qPCR (Garbett et al., 2015), microarray (Hollander et al., 2010; Haramati et al., 2011; Miller et al., 2012; Tapocik et al., 2013; Issler et al., 2014; Lopez et al., 2014a), or candidate-based approaches (Uchida et al., 2008; Baudry et al., 2010; Im and Kenny, 2012; Maheu et al., 2015; Zhang et al., 2015). We chose to use RNA-Seq as it is a powerful technique with several key advantages: single-base resolution, broad dynamic range, low background, and high reproducibility (Finotello and Di Camillo, 2015). We performed small RNA and mRNA sequencing on separate size-enriched fractions of RNA from the same NAc dissections so as to capture the simultaneous expression patterns of miRs and genes. We then applied an *in silico* approach to identify miR-mRNA target interactions.

Our pipeline has several strengths. First, it provides a complete, unbiased snapshot of NAc transcription and post-transcriptional regulation in stressed males and females. Second, we implemented a requirement that miR targets be predicted by both the TargetScan and miRWalk algorithms to increase our confidence in miR-target relationships, as target prediction algorithms can produce false positives (Darnell, 2010). Furthermore, as tissue miR detection does not necessarily denote appreciable repressive activity (Mullokandov et al., 2012; La Rocca et al., 2015), we included an additional layer of anti-correlated miR-mRNA expression networks to support a functional role for identified miRs. Interestingly, we observed a non-canonical positive correlation in a substantial proportion of significant miR-mRNA relationships—61% of miR-targeted genes in females, and 76% of miR-targeted genes in males had a positive correlation with at least one targeting miR. miR activation of targeted genes has been reported (Vasudevan et al., 2007; Henke et al., 2008; Ørom et al., 2008) but is uncommon, and therefore likely represents alternative and indirect regulatory mechanisms not captured by our analysis. Alternatively, as the effect of a single miR on target expression is modest, the net effect of all targeting miRs on a gene may be negative despite some positive miR-mRNA relationships. This observation, coupled with the finding that a majority of miR-targeted genes in males and females were targeted by multiple miRs, highlights the complexity of miR regulation and the utility of coordinated miR activity in fine-tuning gene expression in the brain.

The genome-wide profiles we report corroborate our previous mRNA findings (Hodes et al., 2015a) and extend them to the level of post-transcriptional regulation. Collectively, our results suggest that male mice may be mounting an active, adaptive NAc transcriptional and post-transcriptional response that is unique to the male sex. Several recent studies suggest that a robust transcriptional response contributes to behavioral resilience. Bagot et al. (2016) report that mice resilient to chronic social defeat stress display more differentially expressed genes in NAc compared to controls than do susceptible mice at 48 h post-defeat. We previously found that following SCVS, females show a greater induction of the repressive methyltransferase gene *Dnmt3a* in NAc (Hodes et al., 2015a). Viral knockdown of *Dnmt3a* promoted behavioral resilience, and interestingly produced a greater percent overlap with male transcriptional patterns. Smalheiser et al. (2011) reported evidence that robust miR responses may also contribute to resilience. They observed that rats resilient to learned helplessness show significant regulation of 48% more miRs in cortex than do susceptible rats. Interestingly, the greater proportion of SCVS-regulated genes in males than in females was starker in this analysis than in our previously published RNA-Seq study (60% more genes in males versus 17% in our previous study, Hodes et al., 2015a). This difference may be attributable to the fact that we sequenced only females in estrus and proestrus, and may indicate that females show an especially dampened NAc transcriptional stress response during this period of heightened stress sensitivity.

We chose to sequence samples derived from reproductively intact females in estrus and proestrus due to the hormonal dynamics of the estrous cycle. The rodent estrous cycle consists of four phases: proestrus, estrus, metestrus, and diestrus. Estrogen and progesterone reach peak plasma concentrations during proestrus, decline in concentration throughout estrus, and are present at low concentrations in diestrus (Butcher et al., 1974; Bangasser and Valentino, 2014). Therefore, by studying females in estrus and proestrus, we intended to best capture a period characterized by both hormonal fluctuation and high levels of estrogen and progesterone. Adult women are most susceptible to depression during periods of great hormonal fluctuation, including the postpartum period (Hendrick et al., 1998) and during perimenopause (Cohen et al., 2006; Toffol et al., 2015). Comparatively, female rodents are most susceptible to stress during proestrus—when estrogen and progesterone are high—and exhibit higher plasma corticosterone levels at baseline and in response to stress in proestrus compared to other cycle stages (Viau and Meaney, 1991; Carey et al., 1995; Bangasser and Valentino, 2014). A recent meta-analysis of 311 neuroscience articles found that female rats at any stage of the estrous cycle are not more variable than male rats across behavioral, electrophysiological, neurochemical, and histological measures (Becker et al., 2016), further supporting our rationale for combining females in estrus and proestrus.

Our finding of minimal overlap in genes, miRs, and functional processes between the sexes suggests that the female transcriptional and post-transcriptional stress response is not a blunted male response, but is rather a fundamentally different, sex-specific response. Females engage different NAc molecular processes in response to stress than do males, and this likely has important consequences for behavioral outcome. The sex-specific patterns we observed mirror and greatly expand upon those we have previously reported, now highlighting a role for novel post-transcriptional networks in sex differences in stress responses (Hodes et al., 2015a). We also observed more overlapping enriched pathways and GO terms between total male SCVS and miR-targeted male SCVS gene lists than we did for female counterparts, indicating that miRs may play a more prominent role in stress-responsive molecular processes in males than in females. However, it is important to note that our female functional enrichment analyses were lower powered than those for males as our female gene lists were smaller, potentially exaggerating this effect. Nevertheless, the pronounced sex differences we observed in these complex miR-gene networks may shed light on important mechanistic differences governing sex differences in fundamental NAc neural processes, such as synaptic plasticity (Forlano and Woolley, 2010; Wissman et al., 2012) and neuroactive steroid signaling (Becker, 1999; Almey et al., 2015). Furthermore, our analysis revealed enrichment of pathways in miR-targeted gene lists that may yield future insights into enhanced female stress vulnerability. For instance, Wnt/ß-catenin signaling was an enriched IPA canonical pathway for female miR-targeted genes, and component genes *Dkk2, Wnt7b*, and *Sox5* were downregulated in the female SCVS gene list. As Wnt/ß-catenin signaling is necessary for behavioral resilience to chronic social defeat stress (Dias et al., 2014), downregulation of this pathway may contribute to enhanced female vulnerability to SCVS. Our network analysis also highlights miRs for downstream functional analysis. For example, miR-206-3p—a miR that survived our network analysis in females—was oppositely regulated in males and females following SCVS. Recent studies indicate involvement of miR-206-3p—a known regulator of BDNF signaling—in rat hippocampal response to the rapidly acting antidepressant ketamine (Yang et al., 2014), medial prefrontal cortex-mediated alcohol consumption in rats (Tapocik et al., 2014) and susceptibility to bipolar disorder in human subjects (Wang et al., 2014).

In conclusion, we report that male and female mice exhibit fundamentally different NAc genome-wide responses to SCVS. Differences span both transcriptional and post-transcriptional levels, and highlight a potential role for coordinated mRNA and miR activity in behavioral outcome. These findings inform our understanding of the enhanced susceptibility of women to depression and suggest that, as stress response is strikingly different in males and females, treatment protocols and target identification strategies should take sex specificity into account.

## Author contributions

MP, GH, IP, LS, and SR designed the experiments and pipeline analyses. MP, GH, JF, SG, HA, HC, MF, CM, and MH performed the experiments and acquired data. MP, IP, ZL, ZW, and AM analyzed and interpreted the data. MP and SR wrote the article. All authors read and approved the manuscript.

## Funding

This work was supported by US National Institute of Mental Health grants R01 MH090264 (SR), R01 MH104559 (SR), R01 MH104559 (SR), P50 MHo96890 (SR), R21 MH099562 (SR), F31 MH105217 (MP), T32 MH087004 (MP), T32 MH096678 (MP), and by US National Center for Complementary and Integrative Health grant P50 MH096890 (SR).

### Conflict of interest statement

The authors declare that the research was conducted in the absence of any commercial or financial relationships that could be construed as a potential conflict of interest. The reviewer MF and handling Editor declared their shared affiliation, and the handling Editor states that the process nevertheless met the standards of a fair and objective review.
