# Supplementary material for: Integrative Analysis of Sex-Specific microRNA Networks Following Stress in Mouse Nucleus Accumbens
Source: Front Mol Neurosci. 2016 Dec 23;9:144. doi: 10.3389/fnmol.2016.00144 (PMC5179560; doi:10.3389/fnmol.2016.00144)
Supplement: Supplementary file 1 [file SupplementalInformation.DOCX]

**Supplementary Material**

***Supplementary Figure S1. Estrous cycle monitoring in female mice exposed to SCVS.*** (A). SCVS does not disrupt the estrous cycle. The line graph depicts 4 representative animals and their cycle fluctuations before and after the onset of stress. Mice continue to cycle normally for at least 8 days following the onset of stress. (B). Bar graph illustrating the relative percentage of time that each animal cycles through the various estrous cycle stages. Most mice spend at least 20 to 30 percent of their time in estrus and diestrus.

***Supplementary Table S2. miRs significantly regulated by SCVS in males and females compared to same sex controls.***

| **Males** | | | **Females** | | |
| --- | --- | --- | --- | --- | --- |
| ***Downregulated*** | | | ***Downregulated*** | | |
| **miR** | **log_2_(fold change)** | ***p* value** | **miR** | **log_2_(fold change)** | ***p* value** |
| mmu-miR-130a-5p | -2.00049 | 0.02861 | mmu-miR-1961 | -1.76165 | 0.01654 |
| mmu-miR-6769b-5p | -1.30954 | 0.03507 | mmu-miR-7030-3p | -1.70792 | 0.017094 |
| mmu-miR-6932-5p | -1.18838 | 0.03191 | mmu-miR-6900-5p | -1.16523 | 0.01361 |
| mmu-miR-466n-3p | -1.07800 | 0.03175 | mmu-miR-6979-3p | -1.00952 | 0.00536 |
| mmu-miR-5134-3p | -1.05673 | 0.03656 | mmu-miR-6959-5p | -0.93035 | 0.01347 |
| mmu-miR-344c-5p | -1.03368 | 0.00099 | mmu-miR-449c-5p | -0.73072 | 0.04935 |
| mmu-miR-3547-3p | -1.02133 | 0.00265 | mmu-miR-3064-5p | -0.60314 | 0.00225 |
| mmu-miR-20b-5p | -0.69840 | 0.02169 | mmu-miR-206-3p | -0.59311 | 0.01321 |
| mmu-miR-5121 | -0.61321 | 0.00586 | mmu-miR-669c-5p | -0.4518 | 0.02736 |
| mmu-miR-877-5p | -0.57124 | 0.00912 | mmu-miR-5099 | -0.38512 | 0.01681 |
| mmu-miR-493-3p | -0.55234 | 0.01888 |  |  |  |
| mmu-miR-155-5p | -0.51524 | 0.02851 |  |  |  |
| mmu-miR-466c-5p | -0.42083 | 0.02568 |  |  |  |
| mmu-miR-345-5p | -0.41949 | 0.01215 |  |  |  |
| mmu-miR-187-3p | -0.41789 | 0.00187 |  |  |  |
| mmu-miR-339-3p | -0.38908 | 0.01093 |  |  |  |
| mmu-miR-153-5p | -0.37903 | 0.01151 |  |  |  |
| ***Upregulated*** | | | ***Upregulated*** | | |
| **miR** | **log_2_(fold change)** | ***p* value** | **miR** | **log_2_(fold change)** | ***p* value** |
| mmu-let-7c-1-3p | 0.40377 | 0.03436 | mmu-let-7b-3p | 0.39459 | 0.01726 |
| mmu-miR-451a | 0.40714 | 0.00094 | mmu-miR-1981-3p | 0.41912 | 0.02101 |
| mmu-miR-365-3p | 0.41079 | 0.01251 | mmu-let-7f-1-3p | 0.43917 | 0.01289 |
| mmu-miR-7224-3p | 0.43828 | 0.02425 | mmu-miR-193a-5p | 0.44188 | 0.04242 |
| mmu-let-7e-3p | 0.44169 | 0.01156 | mmu-let-7d-3p | 0.48343 | 0.00493 |
| mmu-miR-1a-3p | 0.44589 | 0.00121 | mmu-miR-503-5p | 0.48372 | 0.00658 |
| mmu-let-7d-3p | 0.45843 | 0.00715 | mmu-miR-98-3p | 0.53471 | 0.00156 |
| mmu-miR-144-3p | 0.46097 | 0.00685 | mmu-miR-7224-3p | 0.5517 | 0.00552 |
| mmu-miR-325-3p | 0.46127 | 0.00233 | mmu-miR-7235-3p | 0.55325 | 0.04590 |
| mmu-miR-672-3p | 0.46271 | 0.04705 | mmu-let-7c-2-3p | 0.56309 | 0.00141 |
| mmu-miR-5099 | 0.48208 | 0.00550 | mmu-let-7a-1-3p | 0.57206 | 0.00155 |
| mmu-miR-133a-3p | 0.48243 | 0.00117 | mmu-miR-1912-3p | 0.63806 | 0.02671 |
| mmu-miR-145a-3p | 0.50507 | 0.00030 | mmu-miR-216a-5p | 1.01792 | 0.02562 |
| mmu-miR-30c-5p | 0.52454 | 0.00056 | mmu-miR-669a-3-3p | 1.05475 | 0.02085 |
| mmu-miR-378d | 0.64896 | 0.03048 | mmu-miR-205-5p | 1.34078 | 0.03217 |
| mmu-miR-144-5p | 0.69028 | 0.00039 | mmu-miR-7092-3p | 2.18364 | 0.00714 |
| mmu-miR-29b-1-5p | 0.74105 | 0.04547 | mmu-miR-3072-3p | 2.2588 | 0.00408 |
| mmu-miR-1912-3p | 0.81205 | 0.00893 | mmu-miR-344h-5p | 2.43715 | 0.00052 |
| mmu-miR-26a-2-3p | 0.82497 | 0.01444 |  |  |  |
| mmu-miR-206-3p | 1.15670 | 0.0001 |  |  |  |
| mmu-miR-7b-3p | 1.16576 | 0.02716 |  |  |  |
| mmu-miR-6939-3p | 1.4258 | 0.03088 |  |  |  |
| mmu-miR-7026-5p | 1.70998 | 0.0097 |  |  |  |
| mmu-miR-181d-3p | 2.08942 | 0.01412 |  |  |  |
| mmu-miR-6926-3p | 2.37142 | 0.00278 |  |  |  |

***Supplementary Table S3. Top 20 genes significantly up- and downregulated by SCVS in males and females compared to same sex controls.***

| **Males** | | | **Females** | | |
| --- | --- | --- | --- | --- | --- |
| ***Downregulated*** | | | ***Downregulated*** | | |
| **Gene** | **log_2_(fold change)** | ***p* value** | **Gene** | **log_2_(fold change)** | ***p* value** |
| *Mir3100* | -3.03433 | 0.00042 | *Serpinb5* | -2.8723 | 0.00665 |
| *AL671140.1* | -2.78301 | 0.00037 | *Chrnb3* | -2.83293 | 0.02854 |
| *Gm8258* | -2.69231 | 0.01242 | *Fermt1* | -2.74172 | 0.00046 |
| *3930402G23Rik* | -2.68671 | 0.0013 | *Tmem184a* | -2.42785 | 0.00301 |
| *2410124H12Rik* | -2.50381 | 0.00986 | *Il28ra* | -2.27804 | 0.00258 |
| *7SK* | -2.49950 | 0.00184 | *Gm12100* | -2.25790 | 0.02879 |
| *snoU13* | -2.47322 | 0.01543 | *AC118193.1* | -2.06149 | 0.01234 |
| *Lcn2* | -2.35153 | 0.04169 | *Gpr44* | -1.97457 | 0.01785 |
| *Akr1c18* | -2.31424 | 0.00022 | *Pla2g10* | -1.94413 | 0.01855 |
| *n-R5s67* | -2.25242 | 0.00973 | *Gm13415* | -1.92972 | 0.03561 |
| *Gpr65* | -2.22001 | 0.01430 | *snoU2_19* | -1.88230 | 0.01255 |
| *Gm12480* | -2.2067 | 0.00838 | *AC164089.1* | -1.83750 | 0.00645 |
| *Gm12747* | -2.18534 | 0.00144 | *5S_rRNA* | -1.801 | 0.00492 |
| *AC124108.1* | -2.13929 | 0.01088 | *0610012D04Rik* | -1.79462 | 0.00223 |
| *Gm11678* | -2.13254 | 0.0144 | *5730412P04Rik* | -1.77746 | 0.04003 |
| *Gm14860* | -2.12619 | 0.00488 | *SNORA48* | -1.75093 | 0.01694 |
| *Atp12a* | -2.09868 | 0.02363 | *Abhd12b* | -1.66442 | 0.02173 |
| *Gm15884* | -2.05284 | 0.02059 | *Lrg1* | -1.63016 | 0.00387 |
| *Hspa1b* | -2.05123 | 0.00007 | *Gm15765* | -1.62412 | 0.02882 |
| *Y_RNA* | -2.02092 | 0.04592 | *Gm17101* | -1.60461 | 0.02326 |
| ***Upregulated*** | | | ***Upregulated*** | | |
| **Gene** | **log_2_(fold change)** | ***p* value** | **Gene** | **log_2_(fold change)** | ***p* value** |
| *Tfap2d* | 3.41226 | 0.00108 | *Klrg1* | 2.18096 | 0.00132 |
| *Chrnb3* | 3.3054 | 0.01482 | *Rps6-ps3* | 1.99043 | 0.0037 |
| *Slc17a7* | 3.24927 | 0.01997 | *AL671140.1* | 1.9765 | 0.00551 |
| *Ighg2c* | 2.89980 | 0.00303 | *Gm12994* | 1.93100 | 0.03364 |
| *AC163292.1* | 2.88457 | 0.00205 | *Gm8104* | 1.82727 | 0.02734 |
| *Dkkl1* | 2.53279 | 0.03668 | *Metazoa_SRP* | 1.82189 | 0.03306 |
| *Trbc2* | 2.42957 | 0.02619 | *Gm12494* | 1.80376 | 0.00033 |
| *Npy6r* | 2.31214 | 0.00202 | *RP23-91L18.2* | 1.80271 | 0.011 |
| *Cplx3* | 2.29744 | 0.02581 | *1700011M02Rik* | 1.71008 | 0.021 |
| *AL808014.1* | 2.16236 | 0.00245 | *Gm16251* | 1.68035 | 0.01289 |
| *Nr4a2* | 2.10573 | 0.04982 | *AL831764.1* | 1.63881 | 0.02572 |
| *E030013I19Rik* | 2.08891 | 0.04659 | *Sh2d1a* | 1.54998 | 0.02954 |
| *Gm2800* | 2.00016 | 0.01231 | *Ace3* | 1.51334 | 0.03710 |
| *Cwh43* | 1.99366 | 0.0289 | *Fam83a* | 1.50039 | 0.01484 |
| *Fcrla* | 1.99284 | 0.01158 | *Gm15817* | 1.49236 | 0.02911 |
| *Adcyap1* | 1.95259 | 0.04364 | *Dennd2d* | 1.4792 | 0.01937 |
| *Haao* | 1.90028 | 0.0019 | *Adtrp* | 1.43257 | 0.0063 |
| *Gm15674* | 1.88896 | 0.01739 | *U6* | 1.41037 | 0.02687 |
| *Gm17334* | 1.85838 | 0.03531 | *SNORA25* | 1.37377 | 0.01353 |
| *Ctgf* | 1.80261 | 0.04106 | *Gm12955* | 1.36178 | 0.01897 |

***Supplementary Table S4. Gene ontology and pathway enrichment analysis results for genes regulated by SCVS in males and females.***

*Pathways/GO terms enriched for SCVS-regulated gene lists in both sexes are listed in bold font.*

| ***Ingenuity Canonical Pathways*** | | | | | | |
| --- | --- | --- | --- | --- | --- | --- |
| ***Male stressed vs. control*** | | | ***Female stressed vs. control*** | | | |
| **Pathway** | | **-log(p*-*value)** | **Pathway** | | | **-log(p-value)** |
| Hepatic Fibrosis / Hepatic Stellate Cell Activation | | 3.22 | Altered T and B Cell Signaling in Rheumatoid Arthritis | | | 2.13 |
| L-serine Degradation | | 2.18 | Cytotoxic T Lymphocyte-Mediated Apoptosis of Target Cells | | | 1.59 |
| **Antigen Presentation Pathway** | | 2.08 | Crosstalk between Dendritic Cells and Natural Killer Cells | | | 1.55 |
| cAMP-Mediated Signaling | | 1.92 | **Antigen Presentation Pathway** | | | 1.53 |
| Unfolded Protein Response | | 1.89 | Ascorbate Recycling (Cytosolic) | | | 1.53 |
| Primary Immunodeficiency Signaling | | 1.56 | MIF-mediated Glucocorticoid Regulation | | | 1.44 |
| G-Protein Coupled Receptor Signaling | | 1.48 | Eumelanin Biosynthesis | | | 1.40 |
| Ketolysis | | 1.39 | Autoimmune Thyroid Disease Signaling | | | 1.37 |
| Bladder Cancer Signaling | | 1.34 | Graft-Versus-Host Disease Signaling | | | 1.34 |
| Thio-molybdenum Cofactor Biosynthesis | | 1.32 | Neuroprotective Role of THOP1 in Alzheimer’s Disease | | | 1.32 |
|  | | | Tetrapyrrole Biosynthesis II | | | 1.31 |
| ***GO Molecular Function Terms*** | | | | | | |
| ***Male Stressed vs. Control*** | | | ***Female Stressed vs. Control*** | | | |
| **GO Term** | **Genes** | **-log(p-value)** | **GO Term** | **Genes** | **-log(p-value)** | |
| **GO:0042165~neurotransmitter binding** | 16 | 5.17 | **GO:0042165~neurotransmitter binding** | 6 | 2.60 | |
| **GO:0030594~neurotransmitter receptor activity** | 16 | 5.17 | **GO:0030594~neurotransmitter receptor activity** | 6 | 2.60 | |
| GO:0005216~ion channel activity | 31 | 3.29 | **GO:0042277~peptide binding** | 7 | 2.10 | |
| GO:0005509~calcium ion binding | 59 | 3.16 | GO:0005125~cytokine activity | 7 | 1.87 | |
| GO:0032403~protein complex binding | 12 | 3.16 | **GO:0008528~peptide receptor activity, G-protein coupled** | 5 | 1.50 | |
| GO:0022838~substrate specific channel activity | 31 | 3.07 | **GO:0001653~peptide receptor activity** | 5 | 1.50 | |
| GO:0022803~passive transmembrane transporter activity | 31 | 2.98 | GO:0050660~FAD binding | 4 | 1.38 | |
| GO:0015267~channel activity | 31 | 2.98 |  | | | |
| **GO:0008528~peptide receptor activity, G-protein coupled** | 14 | 2.89 |  |  |  |  |
| **GO:0001653~peptide receptor activity** | 14 | 2.89 |  |  |  |  |
| GO:0005261~cation channel activity | 23 | 2.73 |  |  |  |  |
| **GO:0042277~peptide binding** | 17 | 2.64 |  |  |  |  |
| GO:0003774~motor activity | 15 | 2.44 |  |  |  |  |
| GO:0022836~gated channel activity | 24 | 2.39 |  |  |  |  |
| GO:0005184~neuropeptide hormone activity | 5 | 2.32 |  |  |  |  |
| GO:0019838~growth factor binding | 10 | 2.32 |  |  |  |  |
| GO:0005516~calmodulin binding | 13 | 2.29 |  |  |  |  |
| GO:0046873~metal ion transmembrane transporter activity | 24 | 2.22 |  |  |  |  |
| GO:0008188~neuropeptide receptor activity | 7 | 2.19 |  |  |  |  |
| GO:0042923~neuropeptide binding | 7 | 2.19 |  |  |  |  |
| GO:0003677~DNA binding | 102 | 2.15 |  |  |  |  |
| GO:0005201~extracellular matrix structural constituent | 6 | 2.00 |  |  |  |  |
| GO:0004983~neuropeptide Y receptor activity | 4 | 1.83 |  |  |  |  |
| GO:0017002~activin receptor activity | 3 | 1.74 |  |  |  |  |
| GO:0043167~ion binding | 201 | 1.73 |  |  |  |  |
| GO:0019842~vitamin binding | 12 | 1.69 |  |  |  |  |
| GO:0005178~integrin binding | 5 | 1.62 |  |  |  |  |
| GO:0008083~growth factor activity | 13 | 1.60 |  |  |  |  |
| GO:0046872~metal ion binding | 195 | 1.52 |  |  |  |  |
| GO:0005179~hormone activity | 11 | 1.51 |  |  |  |  |
| GO:0004222~metalloendopeptidase activity | 11 | 1.49 |  |  |  |  |
| GO:0005227~calcium activated cation channel activity | 4 | 1.49 |  |  |  |  |
| GO:0070279~vitamin B6 binding | 7 | 1.48 |  |  |  |  |
| GO:0030170~pyridoxal phosphate binding | 7 | 1.48 |  |  |  |  |
| GO:0043169~cation binding | 196 | 1.48 |  |  |  |  |
| GO:0003700~transcription factor activity | 46 | 1.33 |  |  |  |  |

***Supplementary Table S5. Significant miR:mRNA networks in females: Up- and downregulated miRs.***

*Only miRs and genes with a negative association are shown.*

| **miR** | ***p* value** | **SCVS-regulated targets predicted by miRWalk and TargetScan** |
| --- | --- | --- |
| ***Downregulated miRs*** | | |
| mmu-miR-206-3p | 0.01415 | *Hoxb9, Tlr4, Arhgap28, Ccbe1* |
| mmu-miR-6900-5p | 0.041405 | *Gdf9, Mtl5, Plekhf1, Chrna2, Sohlh1, Fam83a, Arhgap28, Ccbe1* |
| ***Upregulated miRs*** | | |
| mmu-let-7b-3p | 0.03843 | *Mstn, Pcsk5, Sox5, Prnd, Cxcl13, Ndnf, Myct1, Rreb1, Plekha2, Stard5, Etl4, Mylk3, Adamtsl3, Glt28d2, Htra4, Gm684* |
| mmu-miR-1912-3p | 0.00298 | *Ciita, Dsc2, Gimap1, Lbx2, Sypl2, Pter, Slit2, Sox5, Tbx3, Cd27, Ggt5, Ndnf, Myct1, Rreb1, Crtac1, Arhgap15, 1700028K03Rik, Ifi44, Etl4, Itga10, Slc16a5, Fermt1, A530016L24Rik, Ipcef1* |
| mmu-miR-216a-5p | 0.01284 | *Chrna4, Sypl2, Pax9, Sox5, Serpinb5, Tbx3, Ggt5, Piwil2, Myct1, Rreb1, Esm1, Taf7l, Chrnb3, Mylk3, Fermt1, Adamtsl3, Ipcef1, Fbxl7* |
| mmu-miR-344h-5p | 0.02484 | *Gbp4, Pcsk5, Ggt5, Magix, Piwil2, Aox4, Crtac1, Fam101b, Myzap, Itga10, Slc16a5, Adamtsl3, Ipcef1, Fbxl7* |
| mmu-miR-98-3p | 0.03724 | *Mstn, Pcsk5, Sox5, Prnd, Cxcl13, Dkk2, Ndnf, Myct1, Rreb1, Plekha2, Stard5, Etl4, Mylk3, Adamtsl3, Glt28d2, Htra4, Fbxl7, Gm684* |

***Supplementary Table S6. Significant miR:mRNA networks in males: Downregulated miRs***

*Only miRs and genes with a negative association are shown.*

| **miR** | ***p* value** | **SCVS-regulated targets predicted by miRWalk and TargetScan** |
| --- | --- | --- |
| ***Downregulated miRs*** | | |
| mmu-miR-877-5p | 4.05E-11 | *Adora2b, Cacng1, Cd59a, Cnr1, Kif5a, Slc226, Ptgis, Pvalb, Lynx1, Ermap, Cenpk, Camk2n1, Snx31, Fbxo32, Ecscr, Slc17a7, Slc24a2, Ccdc116, Chrdl1, Slc9a2, Adprhl1, Cplx3, Pla2g4e, Scn4b* |
| mmu-miR-339-3p | 4.90E-4 | *Kif5a, Rapgef4, Chrnb3, Adcy1* |
| mmu-miR-344c-5p | 9.69E-8 | *Chrna4, Adcyap1, Adora3, Cacng1, Cd59a, Cnr1, Kif5a, Pappa2, Ermap, Fbxo32, Tmem144, Tmprss6, Prr5l, Nmnat3, 1300002K09Rik, Ccdc116, Haao, Liph, Acvr1c, Scn4b, Adcy1, Pnpla1, Gm5535* |
| mmu-miR-345-5p | 7.63E-13 | *Cacng1, Itga5, Itgb3, Lynx1, Necab3, Camk2n1, Fam198b, Angel1, Slc24a2, Plekha2, Chrdl1, Chrnb3, Bmf, Rassf3, Ip6k3, Adcy1, Pnpla1, Gm13476* |
| mmu-miR-3547-3p | 1.75E-13 | *Chrna4, Itgb3, St14, Pvalb, Sla, Tfap2b, Lynx1, Rapgef4, Ccl28, Plxdc1, Prr5l, Slc24a2, Ccdc116, Lmod1, Hepacam2, Bmf, Cwh43, Bank1, Tsku, 9930012K11Rik, Acvr1c, Ptpn7* |
| mmu-miR-466c-5p | 1.36E-15 | *Aldh1a1, Cd4, Edn3, Fbn1, Kif5a, Slc22a6, Sla, Pappa2, Lynx1, Ermap, Rapgef4, Ccl28, Tmem178, Fam198b, Prr5l, Arhgap15, Slc24a2, Fgf16, Slc26a5, Chrdl1, Lmod1, Chrnb3, Synpo2, Bmf, Cpne9, Acvr1c, Cntn4, Adcy1, Gm5535, Nlrc5, Trim71, Gm7244* |
| mmu-miR-466n-3p | 2.52E-18 | *Cdh9, Itgb3, Myh3, Npy6r, Satb1, Sla, Chaf1a, Abcg3, Rapgef4, Fbxo32, Samsn1, Rasl11b, 5730508B09Rik, Tmem144, Nmnat3, 1300002K09Rik, 4930558K02Rik, Slc24a2, Fgf16, Chrdl1, Tdrd1, Deptor, Synpo2, Bmf, Etl4, Enpp3, Gcnt4, Clec12a, Liph, Acvr1c, Gm5127, A4gnt, Scn4b, Ccdc87, Gm7244* |
| mmu-miR-493-3p | 8.10E-14 | *Cnr1, Fbn1, Igfbp6, Itgb3, Kif5a, Nr4a2, Slc22a6, Reln, Satb1, Sla, Tnfaip2, Lynx1, Ermap, Ccl28, Tmem178, Tmem144, Plxdc1,*  *Slc24a2, Fgf16, Chrdl1, Tdrd1, Hepacam2, Rassf3, Plekhd1, Myom3, Acvr1c, Clspn, Ip6k3, Ptpn7, Gpr21, Scn4b* |
| mmu-miR-5121 | 6.83E-6 | *Chrna4, Fgf5, Itgb3, Npy6r, Sla, Fbxo32, 5730508B09Rik, Tmem144, Cabyr, Prr5l, Arhgap15, Slc24a2, Ccdc116, Tdrd1, Coro2a, Rassf3, Myom3, Tsku, Adcy1* |
| mmu-miR-5134-3p | 2.69E-6 | *Itk, Pparg, Adprhl1, Scn4b, Pnpla1* |
| mmu-miR-6769b-5p | 4.26E-14 | *Chrna4, Acvrl1, Arhgdib, Cnr1, Edn3, Fbn1, Fhl2, Itga5, Itk, Kif5a, Ky, Tfap2b, Wnt3a, Pappa2, Lynx1, Ermap, Chaf1a, Ccl28, Camk2n1, Snx31, Fbxo32, Cd164l2, Gpx6, Slc24a2, Ccdc116, Plekha2, Coro2a, Synpo2, Bmf, Rassf3, 4931440F15Rik, Plekhd1, Gcnt4, Clec12a, Bank1, Acvr1c, Ptpn7, Adcy1, Pnpla1, Nlrc5, Rhox4a* |
| mmu-miR-6932-5p | 6.30E-5 | *Chrna4, Cd4, Tnfaip2, Lynx1, Chst4, Ermap, Ccl28, Fbxo32, Fam198b, Cabyr, Prr5l, 1300002K09Rik, Slc24a2, Chrdl1, Kdelr3, Plekhd1, Gcnt4, Liph, Myom3, Pla2g4e, A4gnt, 1700015F17Rik, Scn4b, Adcy1* |

***Supplementary Table S7. Significant miR:mRNA networks in males: Upregulated miRs.***

*Only miRs and genes with a negative association are shown.*

| **miR** | ***p* value** | **SCVS-regulated targets predicted by miRWalk and TargetScan** |
| --- | --- | --- |
| ***Upregulated miRs*** | | |
| mmu-miR-7026-5p | 7.04E-19 | *Adra2b, Arc, Atf3, Pcdh15, Bcl2, Calb2, Ccna2, Cdc7, Cldn5, Col11a1, Epb4.1l4a, Fos, Fosl2, Gaa, Gem, Hap1, Htr4, Irs4, Kcnn4, Masp2, Mc1r, Mmp12, Npy1r, Nrp2, Otx2, Furin, Per2, Serpinb2, Plxna1, Prlr, Rasal1, Scn5a, St3gal1, Six1, Slc8a1, Sox1, Tbx3, Thbs1, Vcl, Wnt2b, Spry4, Prnd, Ror2, Pdk4, Banp, Slc5a3, Acrbp, Zfp276, Fxyd6, Ngb, Stk32b, Dedd2, Rreb1, Atp6v1c2, 1700007K13Rik, Lrch3, Tcerg1l, Clec12b, Anxa9, Slc25a18, Dusp11, Lrrc8e, Zkscan3, Slc25a42, Psmb11, Rhoh, Arhgap36, Capsl, 2410124H12Rik, Tmem200a, Adamtsl2, Fndc8, Igsf6, Dnajb1, Nrg4, Gbp6, Plekhg4, Akr1c18, Dennd3, Btaf1, Slc28a3, Prelp, Kcnn3, Rxfp2, Kirrel, Peg10, Trpm8, Zbtb7c, Dot1l, Fam20a, Itga10, Zbbx, Zbtb8b, Tstd1, Qrfp, Acnat1, Szt2, Cyp26b1, Ano5, Tmem45b, Zbtb16, Lancl3, Rxfp3, Pgap1, Ccdc144b, Igsf10, AW551984, Gpr101, Onecut3, Mfrp, Scml4, Ahsa2, Spib, Adam1a, Hist1h4i, Ubn, C530008M17Rik, Amigo3, Dgkk, Slc2a12, Sfmbt2, Ccdc24, Ccdc121, Ccdc36, Gm14137, 1700016G14Rik, 4930469G21Rik* |
| mmu-miR-7224-3p | 9.14E-15 | *Acvr2b, Adnp, Adra2b, Pcdh15, Inadl, Crh, Cyp2b19, Fgf10, Fosl2, Gpr65, Hcn3, Prmt2, Hsf1, Htr4, Mxd3, Myom2, Neb, Nrp2, Oprm1, Per2, Prlr, Ptgs2, Scn5a, St3gal1, Slc8a1, Sox1, Ttn, Xlr3a, Zfp92, Spry4, Banp, Slc5a3, Lpar2, Plxnc1, Tubd1, Sez6l, Zfp276, Fxyd6, Cables1, Ngb, Rere, Rreb1, Slc52a3, Lrch3, Derl3, Lrrc34, Slc25a42, Psmb11, Rhoh, Manf, Igfbpl1, Rassf10, 9930013L23Rik, Fam126a, Ankrd10, Upb1, Dennd3, Btaf1, Adamts6, Slc28a3, Crip3, Igf2bp3, Kcnn3, Kirrel, Peg10, Hps4, Cspp1, Wdr52, Col28a1, 4932411E22Rik, Arid5a, Cbln4, Bcl2l15, Szt2, Fras1, Plekha7, Lancl3, Pgap1, Mrap2, Onecut3, Sdsl, Mfrp, Stk36, Fchsd1, Ubn2, Serac1, Col8a2, BC053749, A230065H16Rik, Ppapdc1a, Hcrtr2, Pnmal2, Ccdc36, Fam196b, Xlr3b, Gm14137* |
| mmu-miR-7b-3p | 1.97E-17 | *Pcdh15, Bcl2, Ccna2, Cdc7, Col11a1, Col4a3, Fbrs, Fgf10, Fosl2, Gjb6, Kcnj5, Mageb3, Mmp12, Nos3, Per2, Prlr, Rasd1, Ret, Rfx1, Slc8a1, Sox1, Tcf7l2, Scn11a, Spry4, Nrk, Sytl4, Pcdh12, Slc5a3, Pmaip1, Asb4, Rere, Fbln7, Sfpq, Lrrc34, Cabp4, Zdbf2, Fam126a, Gbp6, Plekhg4, Btaf1, Dio3, Rad51c, Kcnn3, Peg10, Hspa1a, Grhl1, Igsf1, Unc5d, Cspp1, Samd14, Fam167a, Impg2, Acat3, Acnat1, Samd11, Tnrc18, Pgap1, Pnma3, Gprasp2, Chodl, Ropn1l, Ahsa2, Spib, Wfikkn2, Ubn2, C530008M17Rik, Slc2a12, Greb1l, Mfsd2b, Gm5464, Fam196b, Col6a5* |
| mmu-miR-325-3p | 1.97E-10 | *Chrne, Adra2b, Pcdh15, Bcl2, Zfp36l1, Cbln1, Inadl, Col11a1, Col4a1, Col4a3, E4f1, Efs, Egr2, Eya2, Fat1, Fgf10, Fos, Fosb, Tsc22d3, Gjb6, Lrp2, Leprel2, Hspa5, Hey2, Foxj1, Prmt2, Htr4, Icam1, Ifi47, Inha, Irs4, Mstn, Nfil3, Omp, Pax9, Cfp, Prlr, Rfx1, Rgs16, St3gal1, Slc8a1, Srms, Tac2, Tbx3, Thbs1, Tulp1, Vcl, Gadd45g, Insl5, Insrr, Prnd, Nrk, Cartpt, Sytl4, Fubp1, Lpar2, Irf3, Hgfac, Prickle3, Papolb, Zfp276, Plin4, Piwil1, Sdf2l1, Asb4, Dedd2, Pdlim7, Lrrc18, Rreb1, Atp6v1c2, Casz1, Tcerg1l, Nsun7, Clec12b, Dusp11, Ccpg1, 1110017D15Rik, Syvn1, Myo18b, 1700006E09Rik, 1700026D08Rik, Med25, 6430628N08Rik, Ccdc151, Hhipl2, 9930013L23Rik, Vmn1r65, Otof, Fam126a, Pnck, Akr1c18, Btaf1, Tas1r1, Ppp1r3a, Kcnn3, Kirrel, Peg10, Shkbp1, Grhl1, Igsf1, Kcnk12, Unc5d, Arid5a, Slc39a2, Zbtb8b, Samd14, Baz1a, Xkr6, Zc3h3, Megf6, Samd11, Fras1, Gprc5a, Pla2g4c, Nbeal2, Gpr150, Rxfp3, Zfp760, Pgap1, D630039A03Rik, Gpr101, Pnma3, Gprasp2, Onecut3, Ahsa2, Ubn2, Dpy19l2, C530008M17Rik, Col8a2, Dgkk, Frem3, Sfmbt2, Zfp663, Hcrtr2, Baiap3, Fam196b, Gm14137, Dynlt1f, Gm14308* |
| mmu-miR-365-3p | 3.01E-8 | *Pcdh15, Bcl2, Btg2, Inadl, Cldn5, Dmpk, Eya2, Fosl2, Plxna1, Ptgs2, Rasd1, Ret, Sfrp2, Six1, Slc8a1, Tulp1, Ly6h, Prnd, Lpar2, Stk32b, Sfpq, Anxa9, Slc25a42, Manf, Zfp773, Igsf6, Nrg4, Fam126a, Gbp6, Peg10, Cnksr1, Unc5d, Arid5a, Gpr179, Acnat1, Pla2g4c, Rxfp3, Pgap1, Ptchd2, Esyt3, Ubn2, Gm14461, Zfp663, Pnmal2* |
| mmu-miR-378d | 8.37E-8 | *Adnp, Serpinc1, Smyd1, Cbln1, Ccna2, Col4a3, Fbrs, Fgf17, Fosl2, Slc6a12, Foxj1, Ifi47, Kcnj5, Mc1r, Npy1r, Nrp2, Furin, Plxna1, Plxna3, Ret, Rfx1, Rgs16, St3gal1, Slc8a1, Tbx3, Traf1, Wnt11, Zfp92, Spry4, Ror2, Icosl, Banp, Pcdh12, Rbm14, Zfp276, Piwil1, Midn, Fxyd6, Cables1, Ngb, 2010109I03Rik, Ptgr1, 9530077C05Rik, Slc52a3, Lrch3, Fuz, Anxa9, Dusp11, Lrrc8e, Psmb11, Syvn1, Glt8d2, Igfbpl1, Zfp773, Ccdc151, Fam159b, Igsf6, Dnajb1, Tex13, Hmgb2, Ttll3, Dennd3, Btaf1, Tm6sf2, Igf2bp3, Ppp1r3a, Stard5, Kirrel, Peg10, Hspa1a, Unc5d, Itga10, 4932411E22Rik, Arid5a, Unc45b, Samd14, Gpr179, Megf6, Cyp26b1, Suv420h2, Ano5, Zbtb16, Pgap1, Ptchd2, Pnma3, Onecut3, Stk36, Spib, Esyt3, Fchsd1, Snhg11, Dusp4, Ubn2, C530008M17Rik, Serac1, Sdk1, Dgkk, BC053749, Slc2a12, Mfsd2b, Pnmal2, Ccdc36, Baiap3, Gm14137, Rnf223* |
| mmu-miR-451a | 1.30E-5 | *Epb4.1l4a, Six1, Ttn, Osr1, Wif1, Slc5a3, Pmaip1, Fbln7, Igf2bp3, Hps4, Gpr101, Hist1h2be, Sfmbt2, Hcrtr2, Klf14* |
| mmu-miR-5099 | 0.00014 | *Pcdh15, Klf4, Prlr, Wnt2b, Sgsm1, Slc5a3, Lpar2, Zfp276, Tmem200a, Rassf10, Dnajb1, Grhl1, Hdac4, Wdr52, Fam167a, Pgap1, Kir3dl2, Adam1a, Ubn2, Gm5464* |
| mmu-miR-672-3p | 4.39E-30 | *Adnp, Adra2b, Alox12e, Pcdh15, Bcl2, Brs3, Ciita, Pdia4, Calcr, Cdc7, Inadl, Clcn1, Cxcr4, Col4a1, Col4a2, Col1a2, Dlk1, Dmc1, Efs, Egr2, Fat1, Fbrs, Fgf10, Flt3l, Fosl2, Gaa, Rhpn1, Hap1, Hcn3, Hey2, Hpse, Irs4, Kcnj5, Mc1r, Myo9b, Myom2, Nnat, Npy1r, Nrp2, Omp, Oprm1, Otx2, Pax9, Peg3, Prlr, Ptgs2, Rasal1, Rasd1, Rax, Ret, Rfx2, Rgs16, Scn5a, Slc1a6, Slc8a1, Sox1, Stc2, Stra6, Tap1, Thbs1, Wnt11, Wnt2b, Zfp92, Spry4, Prnd, Nrk, Cartpt, Pdk4, Magel2, Fubp1, Cpsf4l, Sgsm1, Banp, Pcdh12, Slc5a3, Lpar2, Plxnc1, Dnajb5, Sez6l, Midn, Fxyd6, Ngb, Stk32b, Lpin3, Chordc1, Pdlim7, Ints1, Rreb1, Casz1, Ggnbp1, 2700097O09Rik, Slc25a42, Prl8a1, Rhoh, Igfbpl1, 6430628N08Rik, Tmem200a, Hhipl2, Dnajb1, Wdr6, Spon2, Gbp6, Ankrd10, Upb1, Uhrf2, Slc28a3, Rad51c, Prelp, Ppp1r3a, Kcnn3, Stard5, Kirrel, Peg10, Atp12a, Unc5d, Slc39a2, Unc45b, Samd14, Gpr179, Qrfp, Mall, Tshz2, Szt2, Zbtb40, Fras1, Tnrc18, Cyp26b1, Fgd5, Ano5, Fhod1, Lancl3, 5830403L16Rik, Pgap1, Lrrc38, Ptchd2, Myo16, AW551984, Gpr101, Pnma3, Scml4, Spib, Wfikkn2, Fchsd1, Snhg11, Dusp4, Ubn2, Dpy19l2, Serac1, Slc2a12, Sfmbt2, Greb1l, Ccdc24, Pnmal2, Ccdc36, Gm14137, Dynlt1f* |
| mmu-miR-6926-3p | 8.23E-6 | *Arntl, Pcdh15, Smyd1, Calcr, Inadl, Clcn1, Col4a3, Gjb6, Hspa1b, Htr4, Icam1, Kcnj5, Klf4, Mc1r, Mcf2l, Mmp12, Plxna3, Prlr, Sstr5, Tulp1, Prnd, Pcdh12, Slc5a3, Rbm14, Zbtb20, Pdlim7, Rreb1, Slc52a3, Lrfn2, Slc25a42, Zdbf2, Syvn1, Rhoh, Adck4, Mdn1, Ankrd10, Btaf1, Adamts6, Rad51c, Prelp, Stard5, Kirrel, Peg10, Unc5d, Itga10, Col28a1, Samd14, Baz1a, Acat3, Zbtb40, Cyp26b1, Pla2g4c, Rxfp3, F830016B08Rik, Ptchd2, Onecut3, Snhg11, Ubn2, Dpy19l2, Serac1, 1700101E01Rik, Col8a2, Sdk1, Slc2a12, Sfmbt2, Zfp663, Ppapdc1a, Mfsd2b, Xlr4a, Gm14308* |
| mmu-miR-6939-3p | 4.25E-11 | *Pcdh15, Bcl2, Brs3, Ciita, Cbln1, Cdc7, Fosl2, Slc6a12, Hcn3, Hpse, Ifi203, Mc1r, Mstn, Npy1r, Nrp2, Omp, Oprm1, Prlr, Slc8a1, Wnt2b, Xbp1, Zfp92, Scn11a, Fubp1, E2f7, Ahi1, Slc5a3, Dnajb5, Piwil1, Pmaip1, Lpin3, 2010109I03Rik, Dedd2, Sfpq, Pdia6, Lrrc8e, Slc25a42, Zdbf2, Ttc21a, Adck4, Tmem200a, Rassf10, Igsf6, 9930013L23Rik, Nrg4, Fam126a, Dennd3, Btaf1, Prelp, Kcnn3, Kirrel, Hps4, Unc5d, 4932411E22Rik, Impg2, Qrfp, Tshz2, Samd11, Fgd5, Ano5, Pgap1, D630039A03Rik, Lrrc38, Ptchd2, Onecut3, Stk36, Snhg11, Dock6, C530008M17Rik, Greb1l, Zfp663, Fam196b, Klf14, Rnf223* |

***Supplementary Table S8. Gene ontology and pathway enrichment analysis results for miR-targeted genes in males and females.***

*Pathways/GO terms enriched for the entire SCVS-regulated gene list and the miR-targeted gene list for each sex are listed in bold font. Genes with both negative and positive associations with targeting miRs were submitted to the analysis.*

| ***Ingenuity Canonical Pathways*** | | | | | | |
| --- | --- | --- | --- | --- | --- | --- |
| ***Male stressed vs. control*** | | | ***Female stressed vs. control*** | | | |
| **Pathway** | | **-log(p*-*value)** | **Pathway** | | | **-log(p-value)** |
| **Hepatic Fibrosis / Hepatic Stellate Cell Activation** | | 3.43 | **Altered T and B Cell Signaling in Rheumatoid Arthritis** | | | 2.27 |
| **Unfolded Protein Response** | | 2.62 | GPCR-Mediated Integration of Enteroendocrine Signaling Exemplified by an L Cell | | | 1.43 |
| **cAMP-Mediated Signaling** | | 2.15 | Wnt/ß-Catenin Signaling | | | 1.42 |
| **G-Protein Coupled Receptor Signaling** | | 2.12 | TREM1 Signaling | | | 1.42 |
| **Antigen Presentation Pathway** | | 1.86 | Calcium Signaling | | | 1.41 |
| eNOS Signaling | | 1.72 | Leukotriene Biosynthesis | | | 1.39 |
| G-alpha-i Signaling | | 1.63 | Gamma-Glutamyl Cycle | | | 1.34 |
| **Bladder Cancer Signaling** | | 1.58 |  | | | |
| G-alpha-s Signaling | | 1.48 |  |  |  |  |
| **Primary Immunodeficiency Signaling** | | 1.44 |  |  |  |  |
| Prostanoid Biosynthesis | | 1.42 |  |  |  |  |
| VEGF Family Ligand-Receptor Interactions | | 1.37 |  |  |  |  |
| MIF Regulation of Innate Immunity | | 1.36 |  |  |  |  |
| ***GO Molecular Function Terms*** | | | | | | |
| ***Male Stressed vs. Control*** | | | ***Female Stressed vs. Control*** | | | |
| **GO Term** | **Genes** | **-log(p-value)** | **GO Term** | **Genes** | **-log(p-value)** | |
| **GO:0005509~calcium ion binding** | 46 | 3.48 | **GO:0030594~neurotransmitter receptor activity** | 4 | 2.28 | |
| **GO:0032403~protein complex binding** | 10 | 3.12 | **GO:0042165~neurotransmitter binding** | 4 | 2.28 | |
| **GO:0003677~DNA binding** | 79 | 2.85 | GO:0022836~gated channel activity | 5 | 1.57 | |
| **GO:0019838~growth factor binding** | 9 | 2.73 | GO:0005230~extracellular ligand-gated ion channel activity | 3 | 1.53 | |
| **GO:0005201~extracellular matrix structural constituent** | 6 | 2.65 | **GO:0005125~cytokine activity** | 4 | 1.44 | |
| **GO:0005261~cation channel activity** | 17 | 2.26 |  | | | |
| **GO:0005178~integrin binding** | 5 | 2.14 |  |  |  |  |
| **GO:0008528~peptide receptor activity, G-protein coupled** | 10 | 2.10 |  |  |  |  |
| **GO:0001653~peptide receptor activity** | 10 | 2.10 |  |  |  |  |
| **GO:0017002~activin receptor activity** | 3 | 2.03 |  |  |  |  |
| **GO:0005516~calmodulin binding** | 10 | 2.00 |  |  |  |  |
| **GO:0046873~metal ion transmembrane transporter activity** | 18 | 1.97 |  |  |  |  |
| **GO:0042277~peptide binding** | 12 | 1.90 |  |  |  |  |
| **GO:0008083~growth factor activity** | 11 | 1.86 |  |  |  |  |
| **GO:0005216~ion channel activity** | 20 | 1.83 |  |  |  |  |
| **GO:0046872~metal ion binding** | 142 | 1.71 |  |  |  |  |
| **GO:0042165~neurotransmitter binding** | 8 | 1.70 |  |  |  |  |
| **GO:0030594~neurotransmitter receptor activity** | 8 | 1.70 |  |  |  |  |
| GO:0022838~substrate specific channel activity | 20 | 1.70 |  |  |  |  |
| GO:0001871~pattern binding | 10 | 1.70 |  |  |  |  |
| GO:0030247~polysaccharide binding | 10 | 1.70 |  |  |  |  |
| **GO:0003700~transcription factor activity** | 36 | 1.68 |  |  |  |  |
| **GO:0015267~channel activity** | 20 | 1.65 |  |  |  |  |
| **GO:0022803~passive transmembrane transporter activity** | 20 | 1.65 |  |  |  |  |
| **GO:0043169~cation binding** | 142 | 1.59 |  |  |  |  |
| **GO:0005179~hormone activity** | 9 | 1.58 |  |  |  |  |
| **GO:0043167~ion binding** | 143 | 1.52 |  |  |  |  |
| GO:0005520~insulin-like growth factor binding | 4 | 1.51 |  |  |  |  |
| GO:0048407~platelet-derived growth factor binding | 3 | 1.51 |  |  |  |  |
| GO:0016564~transcription repressor activity | 13 | 1.45 |  |  |  |  |
| GO:0004712~protein serine/threonine/tyrosine kinase activity | 3 | 1.42 |  |  |  |  |
| GO:0019887~protein kinase regulator activity | 6 | 1.42 |  |  |  |  |
| GO:0046983~protein dimerization activity | 18 | 1.38 |  |  |  |  |
| GO:0008201~heparin binding | 7 | 1.33 |  |  |  |  |
| GO:0043565~sequence-specific DNA binding | 26 | 1.32 |  |  |  |  |
